# Vessel noise exposures of harbour seals from the Wadden Sea

**DOI:** 10.1038/s41598-023-33283-z

**Published:** 2023-04-15

**Authors:** Dominik André Nachtsheim, Mark Johnson, Tobias Schaffeld, Abbo van Neer, Peter T. Madsen, Charlotte R. Findlay, Laia Rojano-Doñate, Jonas Teilmann, Lonnie Mikkelsen, Johannes Baltzer, Andreas Ruser, Ursula Siebert, Joseph G. Schnitzler

**Affiliations:** 1 Institute for Terrestrial and Aquatic Wildlife Research, University of Veterinary Medicine Hannover, Foundation, Werftstraße 6, 25761 Büsum, Germany; 2 Aarhus Institute of Advanced Studies, Aarhus University, 8000 Aarhus, Denmark; 3 Zoophysiology, Department of Biology, Aarhus University, 8000 Aarhus, Denmark; 4 Marine Mammal Research, Department of Ecoscience, Aarhus University, 4000 Roskilde, Denmark; 5 Norwegian Polar Institute, Fram Centre, 9296 Tromsö, Norway

**Keywords:** Animal behaviour, Ecology, Behavioural ecology, Conservation biology, Environmental impact, Marine biology

## Abstract

The North Sea faces intense ship traffic owing to increasing human activities at sea. As harbour seals (*Phoca vitulina*) are abundant top predators in the North Sea, it is hypothesised that they experience repeated, high-amplitude vessel exposures. Here, we test this hypothesis by quantifying vessel noise exposures from deployments of long-term sound and movement tags (DTAGs) on nine harbour seals from the Wadden Sea. An automated tool was developed to detect intervals of elevated noise in the sound recordings. An assessment by multiple raters was performed to classify the source as either vessels or other sounds. A total of 133 vessel passes were identified with received levels > 97 dB re 1µPa RMS in the 2 kHz decidecade band and with ambient noise > 6 dB below this detection threshold. Tagged seals spent most of their time within Marine Protected Areas (89 ± 13%, mean ± SD) and were exposed to high-amplitude vessel passes 4.3 ± 1.6 times per day. Only 32% of vessel passes were plausibly associated with an AIS-registered vessel. We conclude that seals in industrialized waters are exposed repeatedly to vessel noise, even in areas designated as protected, and that exposures are poorly predicted by AIS data.

## Introduction

Global ship traffic has increased substantially over the last decades leading to an increase in underwater ambient noise levels^1,2^. Underwater noise from ships is now the dominant anthropogenic contributor to the soundscape of the Anthropocene oceans^3,4^. The North Sea is characterised by intense ship traffic and other anthropogenic activities^5,6^. Major shipping lanes pass through the North Sea connecting European ports with the world trade market. The steady increase in offshore installations, in particular offshore wind farms^7^, also contributes to shipping traffic as vessels support the construction and maintenance of these installations. Furthermore, the North Sea is heavily fished, further contributing to high vessel traffic density^8^. However, despite this intense usage, there is a clear lack of knowledge on how often noise-sensitive animals, such as marine mammals, are exposed to vessel noise and what risk this may pose to them.

The harbour seal (*Phoca vitulina*) is one of the most abundant marine mammals in the North Sea, and a large sub-population inhabits the Wadden Sea region^9,10^. Although primarily a coastal species, harbour seals conduct multi-day foraging trips from their coastal haul-out sites in the Wadden Sea into the North Sea^11–13^. The harbour seal is protected under the European Union (EU) Habitats Directive (92/43/EEC) Annexes II and V. Thus, EU member states are required to protect the species’ core areas and designate those sites as Special Areas of Conservation (SACs) under the Natura2000 framework.

Pinnipeds, such as harbour seals, have evolved sensitive in-air and underwater hearing capabilities in keeping with their semi-aquatic lifestyle^14^. Harbour seals have their best underwater hearing between 0.2 and 40 kHz^14,15^. Underwater sound from vessels is broadband with the highest source spectrum levels occurring at low frequencies (below 200 Hz)^4,16^. However, owing to the broadband signature of vessels with cavitating propellers^4,17^, much of the source spectrum of vessel noise overlaps with the best hearing range of harbour seals. Thus, it is relevant to evaluate vessel noise as a potential anthropogenic stressor of harbour seals.

Several studies have estimated the exposure of free-ranging seals to vessel noise by combining satellite telemetry tracks of individual animals with vessel positions broadcast via the Automatic Identification System (AIS) e.g.,^18–20^. Sound propagation modelling is then used to predict the received level of vessel noise and the resulting risk of hearing impairment. This approach is challenging in coastal areas because seals may travel through environments with strongly varying acoustic propagation characteristics. Moreover, only vessels above a certain size and length are required to carry AIS, leading to an unknown underestimation of vessel noise exposure, particularly in relation to smaller vessels^21^. Additionally, fishing vessels may switch off their AIS transponders to hide fishing grounds^22,23^. Despite these important limitations, there have been few efforts to compare modelled exposure rates with field data^18^. There remains therefore a lack of reliable information on the actual exposure rates and noise levels experienced by free-ranging harbour seals.

A direct approach to measure individual exposure is to record noise levels in situ on animals using sound and movement recording bio-logging tags as noise dosimeters^24–26^. Until recently, limited memory and battery capacity constrained these devices to only a few days of recording time. But technological advances now make it possible to record sound continuously for several weeks^27^. These tags additionally contain a GPS sensor that provides accurate locations over the course of the deployment. Additionally, these tags record the three-dimensional movements of the animal, which can shed light on the behavioural context and any changes in behaviour of the animal during noise exposures^24,26,27^.

In this study, we deployed long-duration sound and movement recording tags (DTAGs) on nine harbour seals in the Wadden Sea region of the North Sea to quantify the rate and levels of vessel noise exposure experienced by free-ranging individuals. We used an automated detection approach with a fixed detection threshold to systematically identify high amplitude noise events. These were subsequently classified into vessel noise and other sounds, providing accurate information about exposure rates in the region. Finally, we sought to identify the potential source vessels by combining ship tracking data from the AIS with the locations of the seal during each vessel pass to establish which vessel types were more likely to interact with seals in the area.

## Materials and methods

### Capture and instrumentation

Nine harbour seals were caught at low tide on the Lorenzensplate, a sandbank in the German Wadden Sea (54.44° N, 8.64° E) (Table 1), by deploying and retrieving a seine net with two boats adjacent to the haul-out sites^28,29^. Once the net was hauled ashore, seals were transferred from the large net into tube nets and manually restrained for further sampling and tagging. Each individual was equipped with a DTAG-4 (size: 40 × 33 × 180 mm including flotation, weight: 206 g)^27^. Tags were glued to the dorsal pelage between the shoulder blades, using two-component epoxy resin (Ergo® 7211, Kisling, Switzerland) or superglue (Loctite® 422, Henkel Corp., USA). The DTAGs were programmed to detach from the animals after four weeks and were subsequently relocated by means of an integrated ARGOS transmitter (SPOT 6, Wildlife Computers, USA).

**Table 1:** Overview of nine harbour seals (*Phoca vitulina*) captured in the German Wadden Sea and instrumented with DTAGs in 2016 and 2017.

Animal ID	Capture date	Sex	Age group	Total length [cm]	Weight [kg]
hs16_265b	21.09.2016	Female	Adult	173	77.0
hs16_265c	21.09.2016	Female	Adult	168	61.6
hs17_109a	19.04.2017	Male	Adult	176	69.5
hs17_109b	19.04.2017	Male	Adult	171	95.0
hs17_109c	19.04.2017	Female	Adult	–	78.5
hs17_109d	19.04.2017	Female	Adult	157	52.5
hs17_109e	19.04.2017	Male	Adult	172	83.0
hs17_283a	10.10.2017	Female	Adult	155	62.2
hs17_283b	10.10.2017	Female	Subadult	127	43.0

### Ethics statement

All catches, sampling and tagging were carried out in accordance with relevant guidelines and regulations. All procedures were approved by the responsible governmental ethics committee of the Ministry of Energy, Agriculture, Environment and Rural Areas of Schleswig Holstein, Germany, under animal ethics permit numbers Az V312‐ 72241.121‐19 (70‐6/07) and V244‐3986/2017 (17‐3/14). The Schleswig‐Holstein's Government‐Owned Company for Coastal Protection, National Parks and Ocean Protection granted access to the capture site located within the National Park and UNESCO World Heritage Site ‘Wadden Sea’.

### DTAG data processing

The DTAG-4 consists of a hydrophone, three-axis accelerometers and magnetometers, pressure (i.e., depth) and temperature sensors as well as a GPS. Sound data were stored with a sampling rate of 64 kHz (2016) and 48 kHz (2017) using lossless compression^30^. The GPS uses the snapshot method (similar to Fastloc™,^31^) in which a 64 ms acquisition of the demodulated GPS-band radio signal is stored in memory during surfacings and positions are calculated in post-processing. Movement sensors were sampled at 200 Hz (acceleration), and 50 Hz (magnetometer and depth). In post-processing, movement data were calibrated and decimated to a common sampling rate of 5 Hz using custom tools (http://www.animaltags.org) in Matlab R2018b (The Mathworks, Natick, MA, USA). Additional technical details of the DTAG including on-board processing and detachment method can be found in Mikkelsen et al.^27^.

### Detection of potential vessel passes

**Figure 1:**
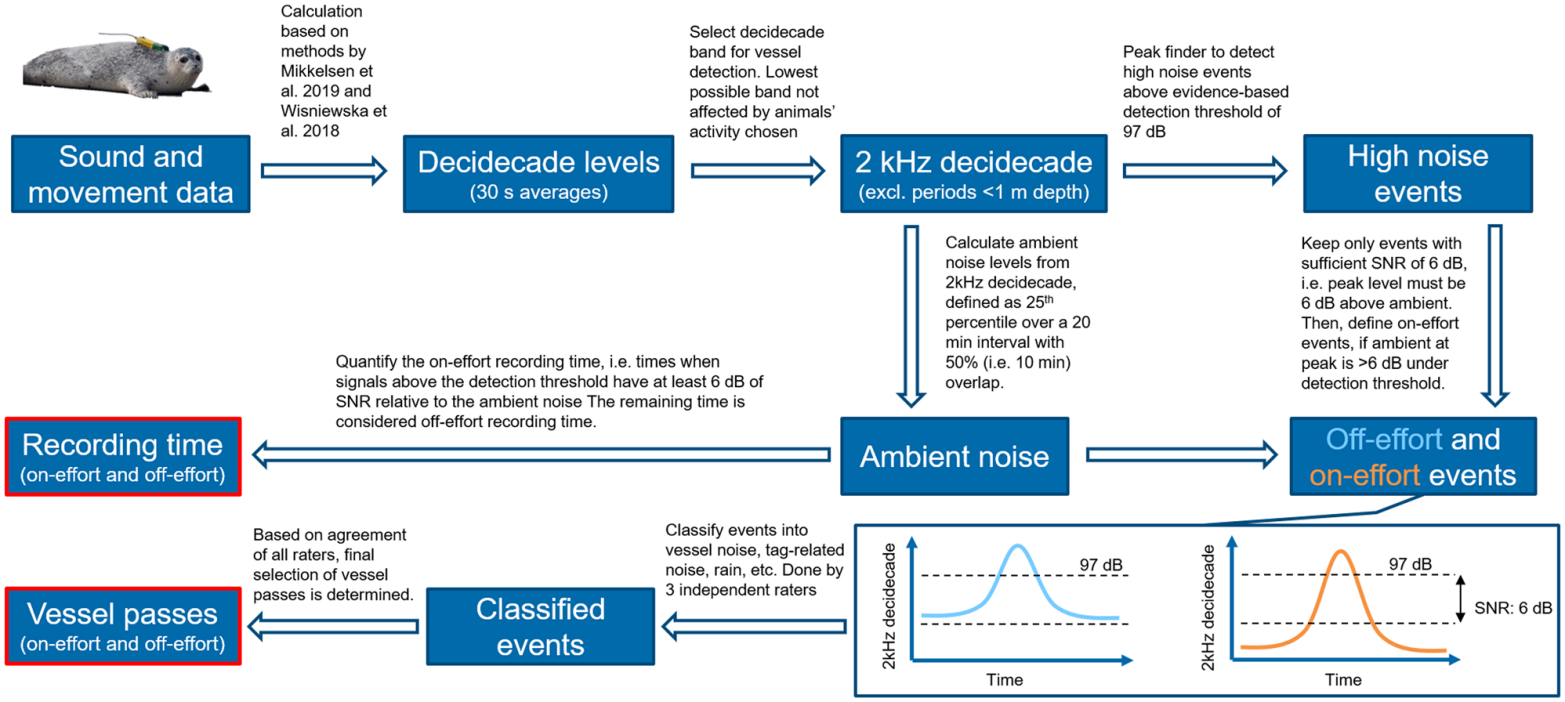
Workflow of the detection and classification of vessel passes, and definition of on-effort and off-effort periods.

As the DTAGs recorded sound and movement data continuously for up to four weeks, an automated method was developed to estimate received sound level and detect transient periods of high amplitude noise in the sound recordings. These high noise events were then classified manually to identify vessel passes. An overview on the workflow for the detection and classification of vessel passes is given in Fig. 1. Sound processing was performed with custom functions developed in Matlab R2018b.

Sound recordings made by an animal-attached device contain sounds from movement of the animal in addition to the ambient underwater sound^26^. Analysis of tag recordings therefore requires different processing steps than do recordings made by a fixed recorder e.g.^32^. To estimate received sound levels, decidecade band levels^33^, also known as third octave band levels, were computed following the methods in Mikkelsen et al.^27^ and Wisniewska et al.^24^. In brief, the sound recording was divided into 30 s consecutive segments, removing segments when the animal was near the surface (i.e., < 1 m depth anytime during the segment). Successive 2048 point Fast Fourier transforms (FFT) (Hann window, 50% overlap) were computed in each segment, resulting in a set of power spectra (1405 for 48 kHz sampled data, and 1874 for 64 kHz data). To avoid transient sounds (e.g., from air bubbles or sudden movements), a subset of these power spectra was averaged to give a single higher-accuracy spectral estimate for each 30 s segment. The spectra for averaging were selected by first summing the power between 3 and 20 kHz from each power spectrum and then selecting the 10% of power spectra with the lowest power in this band. This approach minimises the impact of broadband transients and provides a robust estimate of the continuous environmental noise within each 30 s segment. Decidecade band levels were estimated for each segment by integrating the power in spectral bins that fall into each decidecade band. Finally, power levels were converted into underwater sound pressure levels (dB re 1 µPa RMS) using the calibrated clip level of the tags of 176 dB re 1 µPa.

The resulting decidecade levels include both environmental noise and flow noise generated by the movement of the tagged animal in water^24,34^. At low frequencies, flow noise can exceed ambient noise, making it necessary to select a frequency band for analysis that is high enough to have minimal flow noise but low enough to still have considerable sound energy from vessels. The 2 kHz decidecade band was found to be the lowest frequency band that showed little correlation between sound pressure levels and the activity of the animal, as measured by the log root-mean-squared (RMS) jerk (m s^−3^) (sensu Wisniewska et al.^24^; Supplementary Figs. S1 and S2). This band was therefore used to detect and quantify vessel passes in subsequent steps of the analysis.

A peak finder was applied to the 2 kHz decidecade levels to detect high amplitude events that could potentially be vessel passes (Fig. 1). In passive acoustic monitoring a variable detection threshold, set a predetermined number of Decibels above the ambient noise level, is typically used to detect transient sounds e.g.^35^. As the aim of the present study is to systematically quantify vessel pass exposure rates, we chose a fixed detection threshold across all recordings. This has the advantage of allowing comparison of exposure rates within and across deployments. Using a variable threshold would likely yield more vessel detections, including some faint, distant vessels, but the resulting detection rates depend on the prevailing ambient noise conditions and so cannot readily be compared. Use of a fixed threshold is similar to the use of a strip width in transect sampling surveys from boats or planes^36^. The aim in both cases is not to collect as many detections as possible but to collect them using a standardized effort, which allows robust estimation of encounter rates.

To select the fixed detection threshold, the recordings were initially annotated for all audible vessel passes in a preliminary screening by listening and spectrogram viewing of the sound files. A receiver operating curve (ROC) analysis was then performed based on 2 kHz decidecade levels during annotated vessel passes and levels outside these periods (see Supplementary Methods: ‘Derivation of fixed detection threshold’ for a detailed description). A threshold of 97 dB re 1 µPa RMS in the 2 kHz decidecade band provided the best combination of selectivity and specificity in the annotated data. Applying this detection threshold to the full dataset, any 30 s segment with 2 kHz decidecade band levels above 97 dB re 1 µPa RMS was considered a high noise event. Based on the probable minimum duration of a vessel pass, high noise events closer than 5 min apart were combined into a single event.

Ambient noise levels were estimated from the 2 kHz decidecade band levels by taking the 25th percentile of the 30 s band levels over 20 min intervals with 10 min overlap (Fig. 1). This interval was chosen as vessel passes were usually shorter than 20 min, so that the ambient noise level estimate would be minimally affected by these passes but still reflect the prevailing ambient noise conditions. The estimated ambient noise level varied widely during the deployments and there were occasional periods in which the ambient level approached or exceeded the detection threshold e.g., due to rain or wind. To reliably estimate vessel exposure rates, we defined ‘on-effort’ periods as intervals in the recordings when the ambient noise level was more than 6 dB below the detection threshold, borrowing the terminology from visual surveys (Fig. 1). This rule ensures that on-effort detected high noise events have a signal-to-noise ratio relative to ambient in the 2 kHz decidecade of at least 6 dB facilitating reliable discrimination of vessel noise from other noise sources. Periods with higher ambient noise levels were considered to be ‘off-effort’ and were not included when estimating the rate of vessel encounters (Supplementary Fig. S3).

### Classification of vessel passes

High noise events (both on- and off-effort) were independently classified by three trained raters (D.A.N., L.R.D. and C.R.F.) using listening and spectrogram viewing. A 60 s section around the peak of each event was visualised as a spectrogram (1024 FFT, 50% overlap, Hann window). Raters were offered events for classification in a randomised order and chose one of six classifications: ‘vessel’, ‘potential vessel’, ‘other anthropogenic’, ‘weather/rain’, ‘tag noise’ and ‘unknown’. For events classified as a ‘vessel’, raters had to provide reasoning for their decision, e.g., a Lloyd’s Mirror signature in the spectrogram or rhythmic sound of rotating machinery (see Supplementary Methods: ‘High noise event classification’ for a full description).

Classification results from the three raters were analysed using Cohen’s kappa to assess concordance. The agreement between the raters was very high, with an average agreement of 88% across the 9 tagged seals.

### Association of AIS data with vessel passes

We used AIS data to identify potential vessels giving rise to each classified vessel pass. In Europe, vessels of more than 300 gross tonnage, fishing vessels with a length of more than 15 m, and all passenger ships regardless of size are required to carry AIS transmitters to ensure maritime safety (e.g., ^8^). Via AIS, vessels report their GPS position as well as course, speed, and ship length at regular intervals while underway. Each vessel is identified with a unique Maritime Mobile Service Identity (MMSI) number and transmits a standardised code for its ship type.

The association of each acoustically detected noise exposure with a potential AIS vessel was based on the shape of the noise exposure (i.e., the rise and fall times). A close and/or fast-moving vessel will cause a sound exposure, which rises and falls rapidly, whereas a slow and/or distant vessel will produce a slowly rising and falling sound transient. The expected exposure shape is therefore characterised by the closest approach distance, c in metres, and the speed of the vessel, v in m/s. A propagation analysis suggests that a relevant shape parameter combining these metrics is γ = c/v, which has units of seconds. This parameter is equal to the -3 dB rise and fall time of the exposure, assuming spherical spreading (see Supplementary Methods: ‘Association between vessel noise exposures and AIS data’ for detailed explanation). Thus, γ can be estimated for each candidate vessel from its AIS reports (denoted γ_v_) and, independently, for each noise exposure in the sound recording (denoted γ_n_), enabling an association test.

Although γ_n_ can be estimated in the sound recording from the rise and fall time of the noise exposure, we used a more robust curve fitting procedure. We first computed the 2 kHz octave levels (calculated from the decidecade band levels) in a 10 min window centred around the peak of the vessel pass. These measurements were normalised to the peak exposure power and a quadratic function was fitted to the inverse of the normalised power. The fit of the quadratic function was evaluated by calculating the coefficient of determination, R^2^, and only vessel passes with an R^2^ ≥ 0.7 were used for subsequent analysis (see Supplementary Methods: ‘Association between vessel noise exposures and AIS data’ for more details). The inverse square-root of the first polynomial coefficient was taken as an estimate of γ_n_ for the noise exposure.

Following the calculation of γ_n_ for each vessel pass, we estimated the location of the seal during the event. Assuming that the peak time (i.e., the time of maximum received level during the vessel pass) corresponds to the closest point of approach (CPA), we defined the position of the seal during the vessel event by detecting the GPS locations closest in time before and after the CPA time, and applying a linear interpolation across the two positions. As GPS locations were irregularly sampled and gaps of several hours could occur, we only considered interpolated locations if the closest GPS location was within 60 min of the peak time (see Supplementary Methods: ‘Calculation of seal locations during the peak time of each vessel pass’).

We then identified all AIS vessels reporting within a period of 5 min before and after the peak time of each vessel pass (i.e., 10 min in total) and within a 20 km radius of the seal’s interpolated location at the CPA time. This radius was chosen based on the vessel noise detection threshold and the predicted received levels from different ship types (see Supplementary Methods: ‘Ship sound propagation loss to determine radius for AIS data matching’). This resulted in a set of candidate vessels, for which we computed γ_v_ based on its closest reported position to the seal's location at peak exposure, and its median speed over a 10 min time window centred on the peak exposure time.

The shape parameters, γ_v_ and γ_n_, derived from AIS and the sound recording should coincide if the vessel is the correct source of the exposure. Assuming that the closest vessel is usually the source of the exposure, a linear relationship was established between γ_v_ and γ_n_ pooling all exposures (R^2^ = 0.28, p < 0.001). This model was then used to identify which AIS vessel was likely the actual source vessel for each exposure: AIS vessels that fell within 2 times the standard deviation around the linear regression line were considered ‘likely source vessels’ (see Supplementary Methods: ‘Association between vessel noise exposures and AIS data’ for more details).

AIS vessels were classified into ship type categories based on their AIS codes (see Supplementary Table S1 for translation of AIS codes into ship types). Additional information on each AIS vessel was acquired from publicly available online databases (www.myshiptracking.com, www.vesselfinder.com and www.marinetraffic.com; URLs accessed on 02.03.2022).

## Results

### Spatial distribution of harbour seals

**Figure 2:**
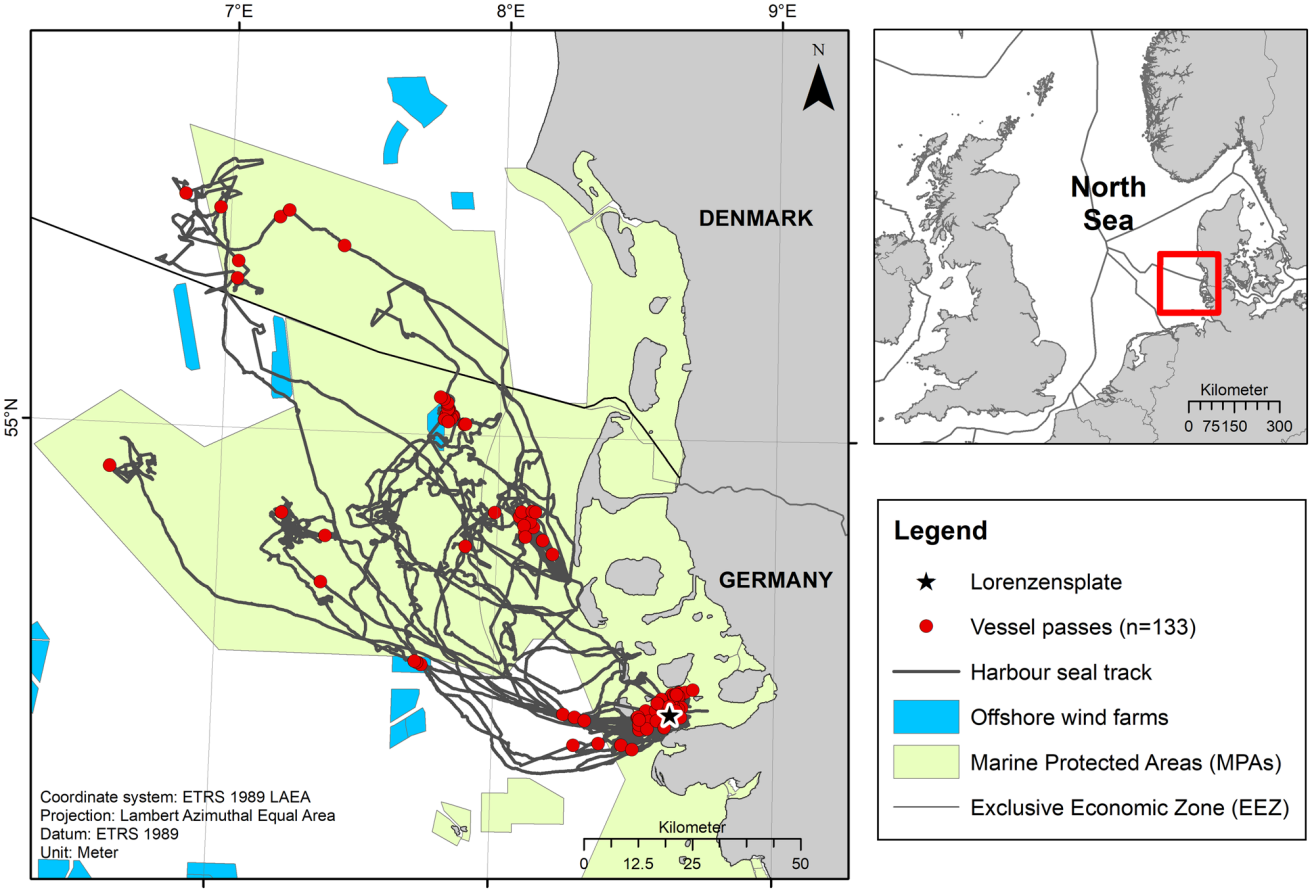
Tracks of harbour seals (n = 9) in the North Sea. The red dots illustrate the locations of high level vessel passes during on-effort periods (n = 133). The tagging site Lorenzensplate is indicated by a black star. Harbour seals were tagged in three catches over two consecutive years. The map was created using ESRI ArcGIS, version 10.5.

Seven of nine individuals made one or two multi-day offshore trips into the North Sea and returned to the Wadden Sea for haul-out (Fig. 2, Vance et al.^11^). Two individuals (hs17_109c and hs17_109d) performed only short inshore trips in the tidal areas of the Wadden Sea for the whole deployment duration. Most seals showed a high degree of site fidelity and regularly returned to the Lorenzensplate to haul-out (Fig. 2).

The tagged harbour seals spent 89 ± 13% (mean ± SD) of their time in MPAs, i.e., the National Park and UNESCO World Heritage Site ‘Wadden Sea’ and the Special Areas of Conservation (SACs) ‘Sylt Outer Reef’ and ‘Southern North Sea’ (Fig. 2). Occasionally, seals passed offshore wind farms, but individuals spent relatively little time in the vicinity of these sites (Fig. 2).

### Exposure to vessel passes

The detection process yielded 560 high noise events, of which 321 (57.3%) were classified as vessel passes. 133 out of the 321 vessel passes occurred during on-effort periods, i.e., when ambient noise was > 6 dB below the detection threshold. The remaining 188 vessel passes were disregarded in the exposure rate calculation as they occurred during high ambient noise periods (Table 2). On-effort periods comprised a total of 735 h (i.e., 39% of the pooled recording times).

**Table 2:** Overview on deployment duration, recording times, number of detected vessels and exposure rate to vessels per day for each tagged individual.

Animal ID	Deployment duration (days)	Total recording time (h)	Recording time on-effort (h), (Proportion of total recording time in %)	Recording time off-effort (h), (Proportion of total recording time in %)	No. of vessels on-effort	Vessel exposure rate (No. of on-effort vessels per day)
hs16_265b	10.8	124	64.5 (52.0%)	59.5 (48.0%)	17	6.3
hs16_265c	21.5	275.5	89.6 (32.5%)	185.9 (67.5%)	22	5.9
hs17_109a	15.7	172.7	60.2 (34.9%)	112.5 (65.1%)	6	2.4
hs17_109b	7.3	99.7	28.8 (28.8%)	71 (71.2%)	4	3.3
hs17_109c	9.3	113.4	49.3 (43.4%)	64.2 (56.6%)	9	4.4
hs17_109d	25.3	259	120.5 (46.5%)	138.5 (53.5%)	17	3.4
hs17_109e	26.6	294.6	95.1 (32.3%)	199.5 (67.7%)	17	4.3
hs17_283a	23.7	273.4	110.7 (40.5%)	162.7 (59.5%)	30	6.5
hs17_283b	21.6	266.4	116.7 (43.8%)	149.7 (56.2%)	11	2.3

The total recording time is the effective time when underwater sound levels could be measured, i.e., excluding haul-out, surfacing and outage periods. The recording time on-effort represents the times when the ambient noise in the 2 kHz decidecade was > 6 dB below the detection threshold of 97 dB re 1µPa. The recording time off-effort represents the times when ambient noise was above 91 dB re 1µPa, i.e., noise conditions are not sufficient to reliably detect a vessel. Only vessels detected during on-effort periods are used to calculate exposure rates.

**Figure 3:**
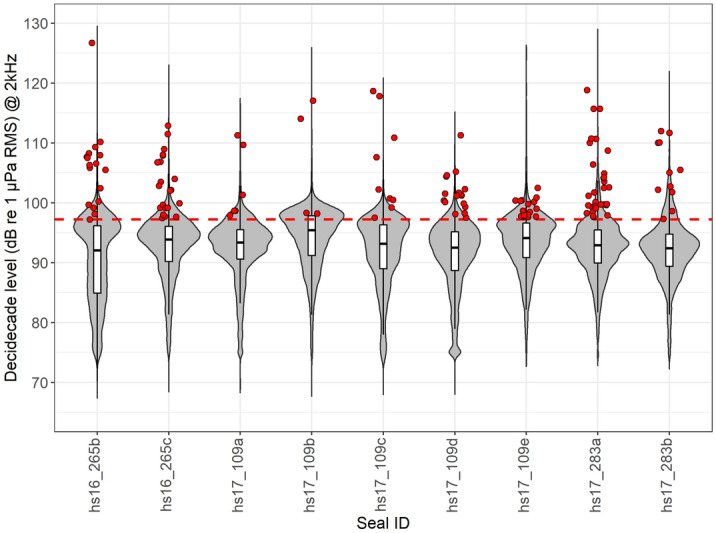
Distribution of 2 kHz decidecade levels (dB re 1 µPa RMS), i.e., the RMS sound pressure level in the 2 kHz decidecade band, for each seal shown as violin plots. The small boxplots within the violins indicate the median and interquartile range of the distributions. The red dashed line illustrates the threshold (97 dB re 1 µPa) for vessel detections. The red points represent the maximum received levels of each vessel pass during on-effort periods (n = 133); the points are randomly spread horizontally to increase visibility.

**Figure 4:**
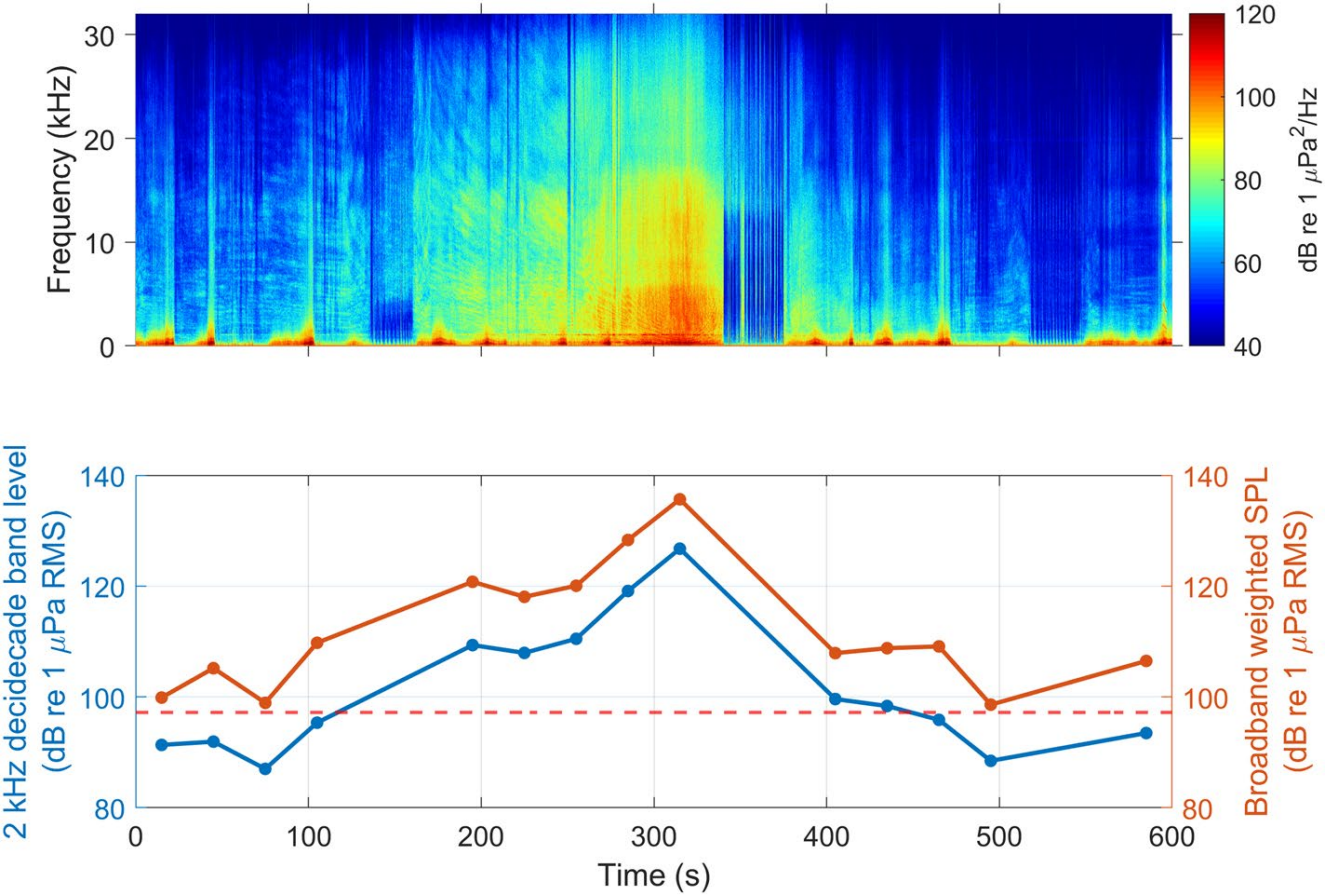
Vessel pass with the highest maximum 2 kHz decidecade received level in the study. The top image shows a spectrogram of the power spectral density (PSD, i.e., power per 1 Hz band). The vessel noise in the recording is interrupted multiple times due to surfacing of the seal. The bottom image shows the corresponding 2 kHz decidecade band levels (blue line), computed as 30 s averages as described in the text, as well as broadband weighted sound pressure levels (SPL; orange line) from 500 Hz to 20 kHz following the frequency weighting for phocid seals in water (PCW) by Southall et al.^37^. The red dashed line illustrates the 97 dB detection threshold used to detect high noise events in the 2 kHz decidecade band.

On average, seals were exposed to 4.3 ± 1.6 vessel passes per day (Table 2) during on-effort periods. The maximum decidecade received levels, i.e., the root mean square (RMS) sound pressure levels in the 2 kHz decidecade band, varied across vessel exposures with an average of 103 ± 6 dB re 1µPa RMS @ 2 kHz (Fig. 3). The highest received level recorded during an on-effort vessel pass was 127 dB re 1µPa RMS @ 2 kHz (30 s average) (Fig. 4). Harbour seals encountered vessels both during their offshore trips and while inshore in the Wadden Sea (Fig. 2).

Most vessel encounters occurred within MPAs (on average 93 ± 7%). By pooling vessel passes and on-effort periods from all individuals, vessel exposure rates seemed to be higher within MPAs (4.5 vessel passes per day) compared to outside MPAs (3.0 vessel passes per day). However, given the small aggregate time spent by seals outside of MPAs, this difference may be unreliable.

### Association between vessel noise exposures and AIS data

The shape parameter γ_n_ was calculated for all 321 vessel passes (both on-effort and off-effort) from the noise exposures. Based on the goodness-of-fit of the quadratic function (R^2^ ≥ 0.7) and the estimation of a location of the seal at the peak time (see Supplementary Methods for more details), 148 vessel passes were retained for the association with AIS data.

**Figure 5:**
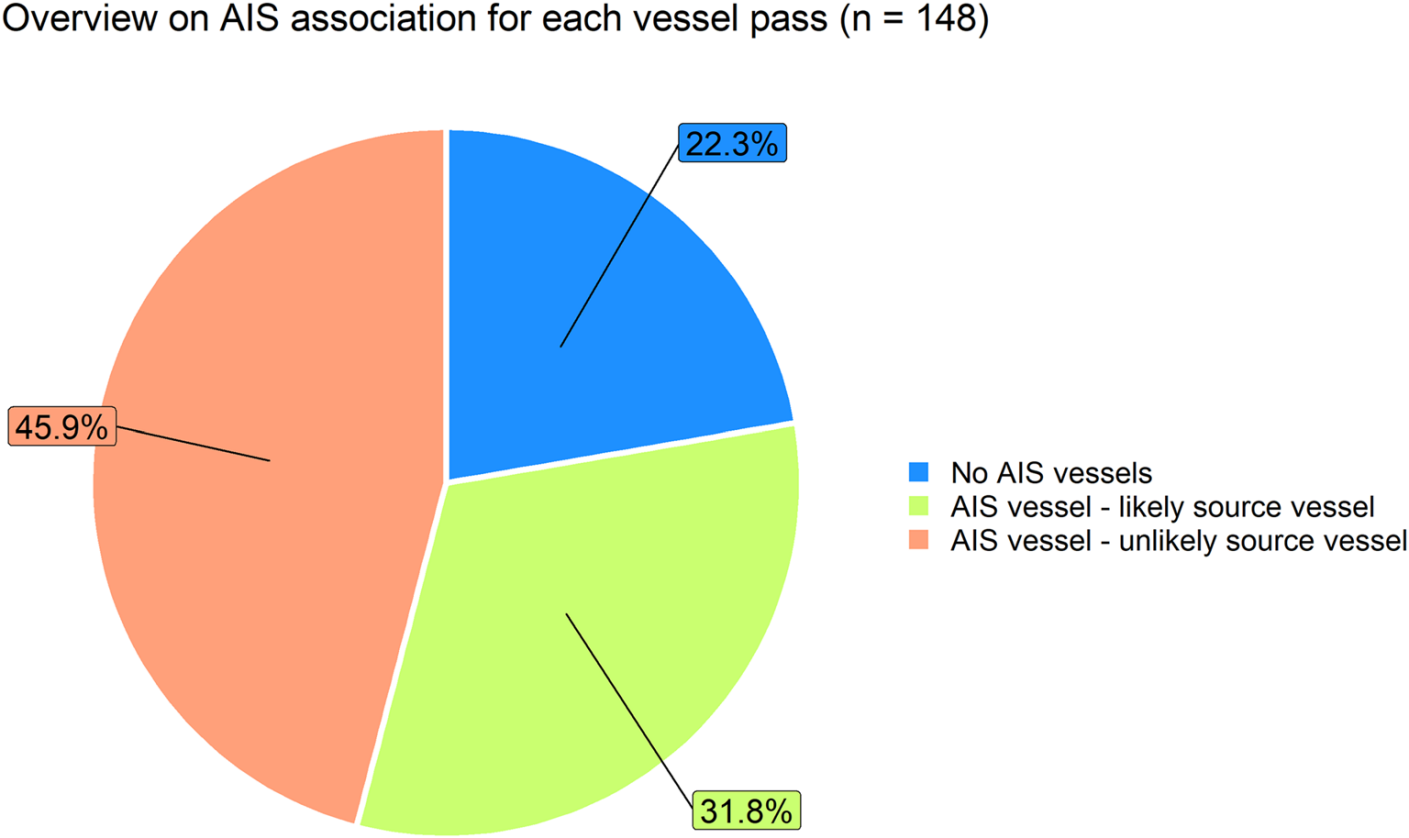
Overview of the association between recorded vessel noise exposures (n = 148) and AIS data. Blue shows the proportion in which either no AIS vessel or only stationary vessels were present in a 20 km radius. Light green illustrates the proportion of exposures in which an AIS registered vessel was likely the actual source of the noise exposure based on consistent shape parameters, whereas orange indicates the proportion of exposures where the recorded noise exposure cannot be attributed to any of the present AIS vessels.

In 33 of 148 vessel passes (22%), either no or only stationary AIS registered vessels were present within 20 km of the seal and within a 10 min time window around the time of peak exposure. In the remaining 115 cases (78%), at least one AIS vessel was travelling within 20 km around the seal (Fig. 5).

In most cases, more than one AIS vessel was present in the vicinity of the seals, with a maximum of 24 vessels. To assess which, if any, of the AIS vessels near the seal might have caused the exposure, we compared the shape parameter of the noise exposure, γ_n_, with the expected shape parameter for each vessel, γ_v_, derived from the AIS data. In 47 exposures (32% from 148 vessel passes), at least one AIS vessel had a γ_v_ value consistent with the exposure γ_n_, thus being the likely source of the noise exposure. In 123 cases (46% from the 148 vessel passes), no AIS vessel had a speed and estimated approach distance consistent with the shape parameter derived from the noise exposure in the DTAG sound data (Fig. 5).

**Figure 6:**
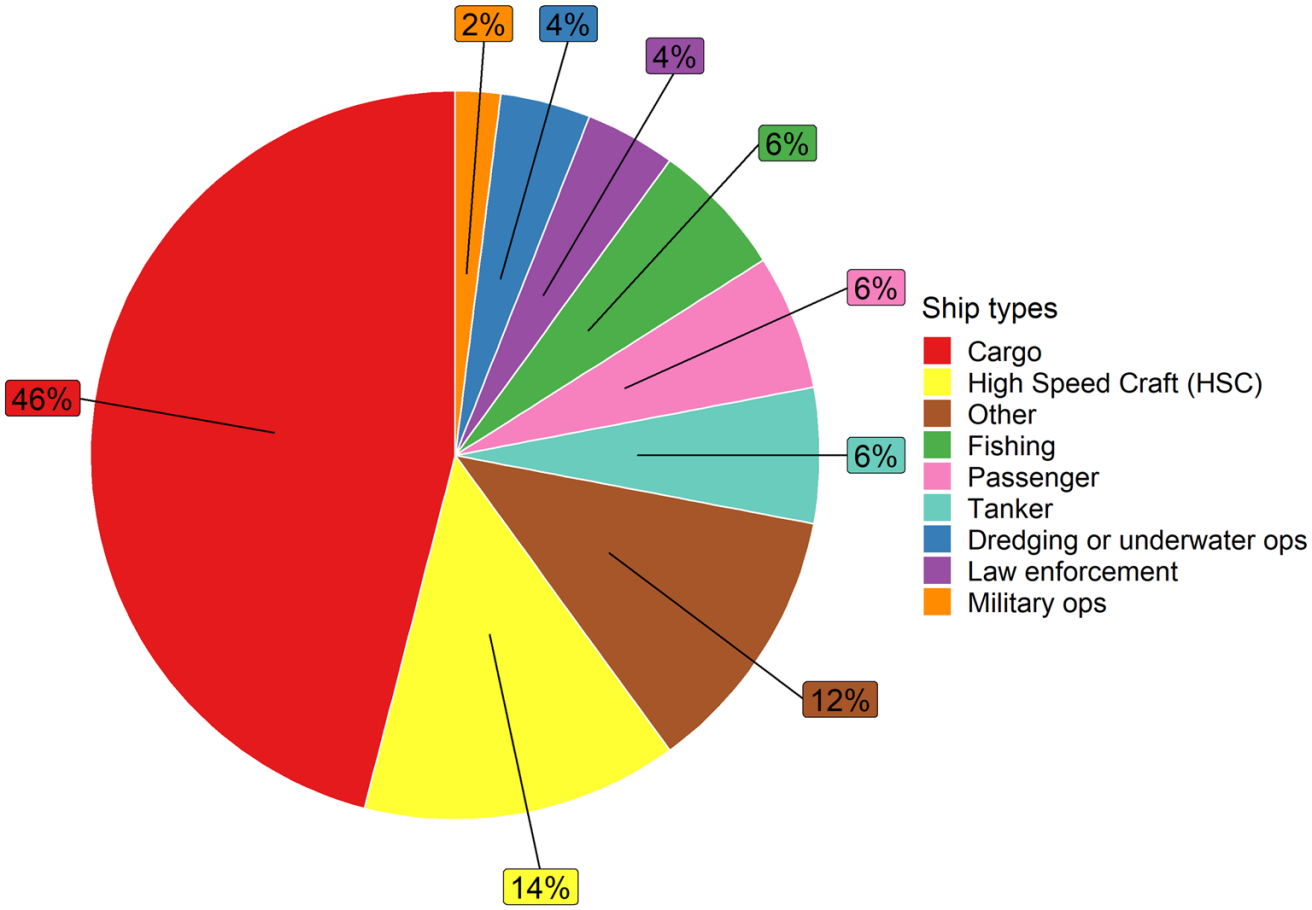
Pie chart of ship types based on those vessel passes where the likely source vessels could be identified from the AIS data (n = 47).

Based on the 47 exposures in which the source vessels could likely be identified from the AIS data, harbour seals encountered cargo ships most frequently (23 vessel passes; 46%) (Fig. 6), followed by high-speed crafts (7 vessel passes; 14%) and other vessels (6 vessel passes; 12%), a category which includes research vessels and offshore support ships. (Fig. 6). Several encounters with fishing vessels, tankers and passenger ships were also detected.

## Discussion

Harbour seals are central place foragers that partition their time between resting at their central place (i.e., their ‘colony’ on land) and searching for food at sea^11,12,38^. The seals tagged in this study showed the same behavioural patterns, with most of the seals travelling between inshore areas in the Wadden Sea and offshore areas in the North Sea. By using a systematic detection and classification approach, we find an average exposure rate of 4.3 vessel exposures per day for seals at sea. This exposure rate only includes medium to high noise exposures owing to the 97 dB re 1 µPa (2 kHz decidecade) threshold used for detection, and therefore does not account for less intense exposures from quieter or more distant vessels. Nevertheless, quantifying the exposure rate towards a given stressor, such as high amplitude vessel passes, is an essential first step to inform models on the consequences of disturbance and hence, improve our understanding on the impacts of anthropogenic disturbances on marine mammal populations^39^.

Sound recordings made directly on the harbour seals allowed us to quantify both vessel exposure rates and received noise levels. Maximum received levels during the vessel passes were on average 103 dB re 1µPa RMS in the 2 kHz decidecade band and the maximum received level of any vessel pass was 127 dB re 1µPa RMS in the same band. The corresponding broadband levels of these exposures could not be directly measured in this study due to the variable low frequency flow noise inherent in on-animal sound recordings. The hearing-weighted broadband levels would be substantially higher than the levels in the 2 kHz decidecade band because vessels produce greater sound energy at low frequencies (Fig. 4, MacGillivray and de Jong^16^). Although it may be possible to predict hearing-weighted sound levels to some degree from the higher-frequency band levels that can be measured with tags, the strength of the tag-based dosimetry approach used here is that it provides a direct measure of the vessel noise exposure rate to individual animals.

Alternative approaches to quantify vessel exposure rates and received noise levels combine animal tracking data with AIS reports of vessel movements and rely on source level predictions and sound propagation modelling, both of which require a range of assumptions (e.g., choice of sound propagation and vessel source level models, resolution of seal and ship locations, environmental data, etc.)^18–20^. In addition, these predictive studies are dependent on the completeness of AIS data and cannot account for vessels without AIS which can be numerous in coastal areas^21^. In our study a majority of vessel noise exposures could not be reconciled with an AIS vessel within a 20 km radius around the seals. Only 32% of the vessel noise events were associated with an AIS vessel that had a speed and approach distance consistent with the shape of the noise exposure. The low predictive potential of AIS data for the recorded vessel noise exposures is surprising, but could be explained by (1) spatial and temporal gaps in the AIS data^40^, (2) the presence of small vessels without AIS^21^, and (3) AIS vessels that have switched off their AIS transmitter, e.g., fishing vessels potentially obscuring illegal fishing activities^22,23^. Our method for associating AIS reports with noise exposures may also produce some errors, e.g., due to the complex and variable sound propagation in shallow water environments as well as the movements of the seal during the exposure, and further validation is needed. However, the profound lack of a simple one-to-one connection between audible vessel passes and AIS vessels found here, highlights the risk of substantial errors when predicting animal noise exposure based only on the AIS vessels in the vicinity of their track lines.

Although a large proportion of vessel noise exposures could not be attributed to an AIS vessel, the matches between detected vessel passes and AIS vessels indicate that tagged harbour seals encountered a variety of ship types in line with the diverse human use of the North Sea^6^. Amongst AIS vessels, tagged harbour seals most often encountered cargo ships (46%) and high-speed crafts (14%). Both usually travel along predefined routes between ports or between the port and offshore installations, such as offshore wind farms. High-speed crafts are typically involved in the maintenance of offshore installations, transporting crew and material. The expansion of offshore renewable energy will foster an increase in service traffic, potentially leading to more vessel exposures in the future. The frequent encounters with cargo ships and high-speed crafts, as well as the encounters with fishing vessels and passenger ships, suggest that attractive habitats for harbour seals are located in close vicinity to major shipping routes and fishing grounds. Thus, harbour seals may face a trade-off between favourable foraging or resting grounds and frequent exposure to noise from passing ships.

Studies on the effects of vessel noise on seals are scarce, but behavioural responses have been anecdotally reported from exposure at sea and on land^27^. Thresholds for behavioural responses, such as a cessation of feeding activities leading to missed foraging opportunities, are critically needed to assess the ecological consequences of vessel noise on harbour seals. The methodology and tools developed in this study for automatic vessel pass detections and AIS allocation provide a critical step forward, as manual analysis is not feasible for the long-duration recordings needed to study chance exposures. Quantifying the exposures to vessel noise with long-term sound and movement tags allows us to study changes in behaviour as a next step and are therefore especially useful in evaluating the cumulative impact of vessel exposures.

Harbour seals in the present study spent most of their time in MPAs. Multiple ship-based anthropogenic activities are allowed in the MPAs: shipping lanes pass through them, one operating offshore wind farm (‘Butendiek’) is located within the SAC Sylt Outer Reef and commercial fishing activities take place in these protected areas with little regulation^41,42^. The fundamental purpose of MPAs is to preserve the habitat and provide refugia for sensitive species in order to stabilise population levels. The protection conferred by these sites should therefore extend to anthropogenic disturbances that impact biologically important behaviours (e.g., resting, foraging, and reproducing). In this study we highlight the potential for harbour seals to be repeatedly exposed to high amplitude vessel noise within protected sites. If such exposures evoke energetic behavioural responses, their frequent repetition over extended periods could have consequences for the individual fitness of seals, which in turn may impact the conservation status of harbour seal populations within these protected sites if appropriate management measures to reduce vessel exposures and noise are not taken.

## Conclusion

The present study quantified exposure rates of nine free-ranging harbour seals to moderate-to-high amplitude vessel noise in the Wadden Sea and adjacent North Sea. Using a systematic approach to detect vessel noise in long-term on-animal acoustic recordings, we demonstrate that harbour seals are on average exposed to 4.3 high-noise-level vessel passes per day. Concurrent AIS data enable association of noise exposures with specific vessels in some cases (32%), providing insights into which vessel classes contribute most to the levels received by animals. However, in the major proportion of cases (68%)—either because no AIS vessel was present or the recorded noise exposure could not plausibly be attributed to any of the present AIS vessels—we were unable to associate vessel noise exposures with an AIS-registered vessel, highlighting that animal noise exposure estimation based solely on AIS data can be prone to substantial errors.

Most vessel encounters took place within MPAs highlighting the potentially extensive anthropogenic use of these areas despite their protected status. The quantification of vessel noise exposure rates is the first step in assessing the ecological relevance of vessel noise to seals, which may be particularly vulnerable due to their good low frequency hearing. This information will be essential to evaluate the cumulative physiological and ecological impact of vessel passes on harbour seals to inform appropriate mitigation measures.

## Supplementary Information

Supplementary Information.

## Data Availability

The datasets generated and analysed during the current study are available in the Dryad repository under 10.5061/dryad.mkkwh714m.
