# Supplementary material for: Vessel noise exposures of harbour seals from the Wadden Sea
Source: Sci Rep. 2023 Apr 15;13:6187. doi: 10.1038/s41598-023-33283-z (PMC10105764; doi:10.1038/s41598-023-33283-z)
Supplement: Supplementary file 1 — Supplementary Information. [file 41598_2023_33283_MOESM1_ESM.pdf]

Supplementary Figure S1

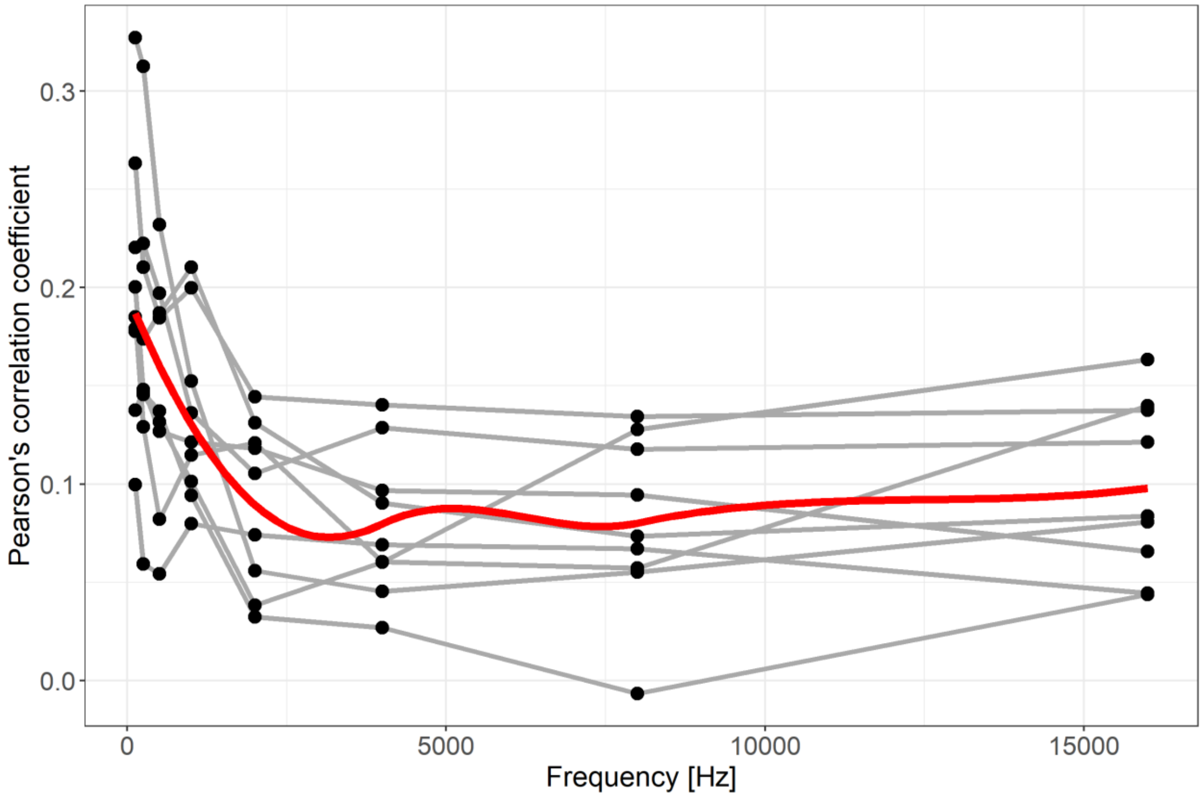

Supplementary Figure S1: Correlation between sound levels and the log RMS jerk, quantified by the Pearson's correlation coefficient, for eight decidecade bands (125 Hz - 16 kHz). A high correlation coefficient indicates that sound levels in the band are strongly related to activity, e.g., due to flow and propulsion noise. Black points connected with grey lines indicate the results for each of the nine seals. The red curve is a loess smoother (span = 0.8) based on the nine measurements. The correlation is higher at lower frequencies, indicating activity-dependent flow noise. The correlation decreases with frequency and becomes minimal at 2 kHz. This decidecade band was therefore selected for the semi-automated vessel detection procedure in the main text.

Supplementary Figure S2

hs16_265b

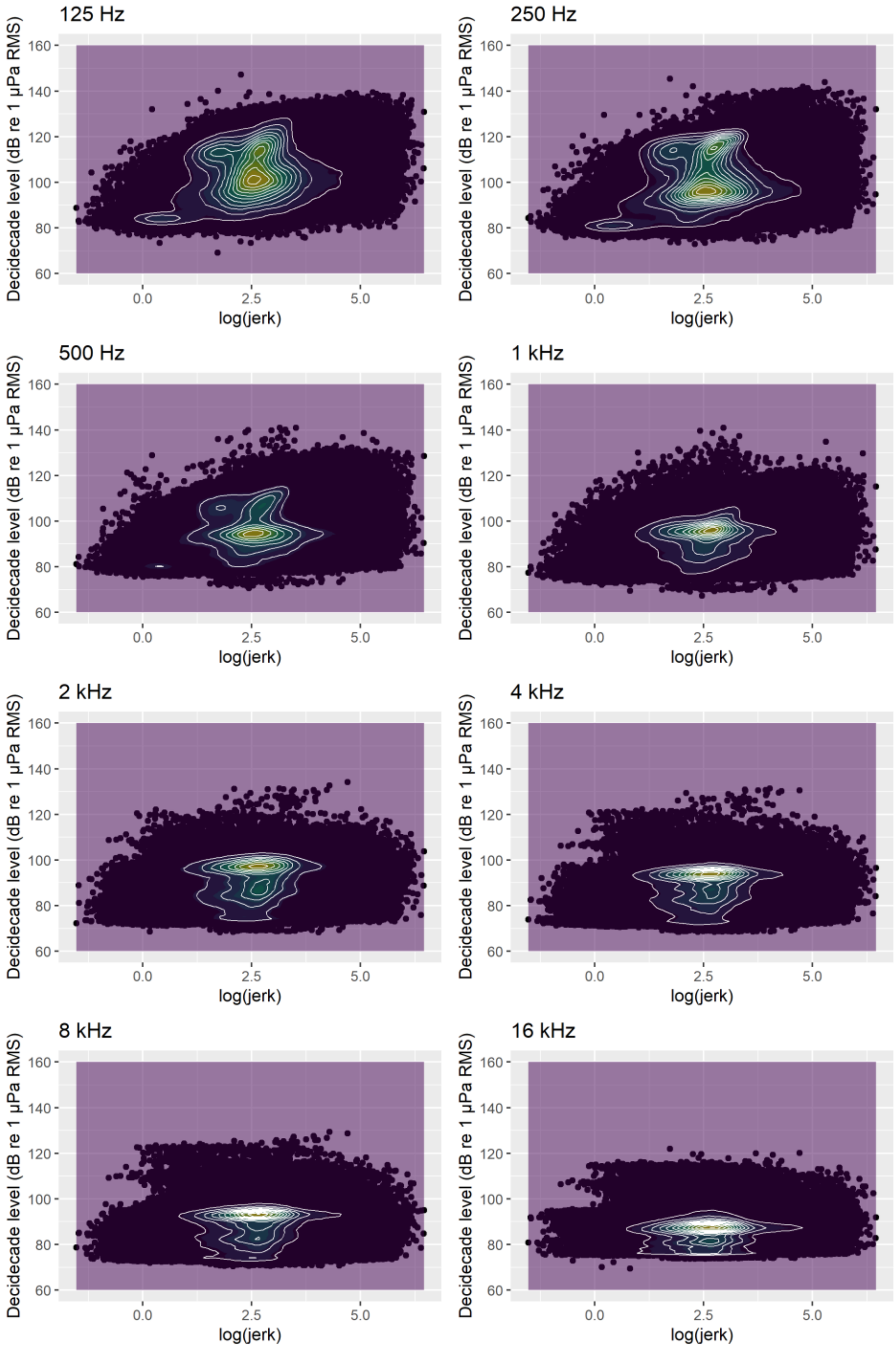

Supplementary Figure S2: Sound levels in eight different decidecade bands in relation to an acceleration-based measure, the log RMS jerk (see Wisniewska et al., 2018 for detailed method description). Sound levels and jerk values are shown as 1 s averages. The data points are overlaid by kernel density estimates shown by contour lines and different colours. Each page shows data from one animal.

hs16_265c

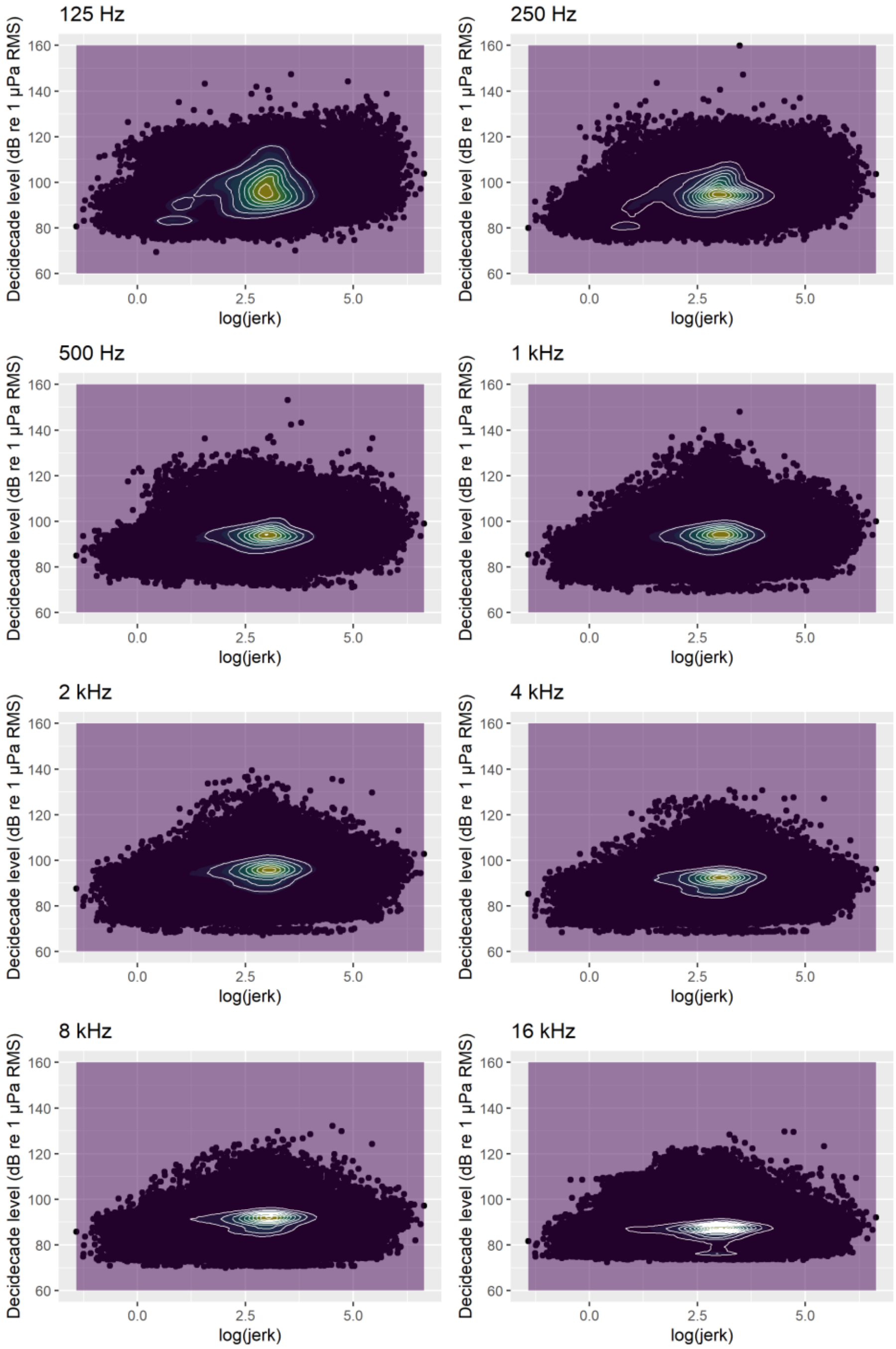

Supplementary Figure S2 (continued)

hs17_109a

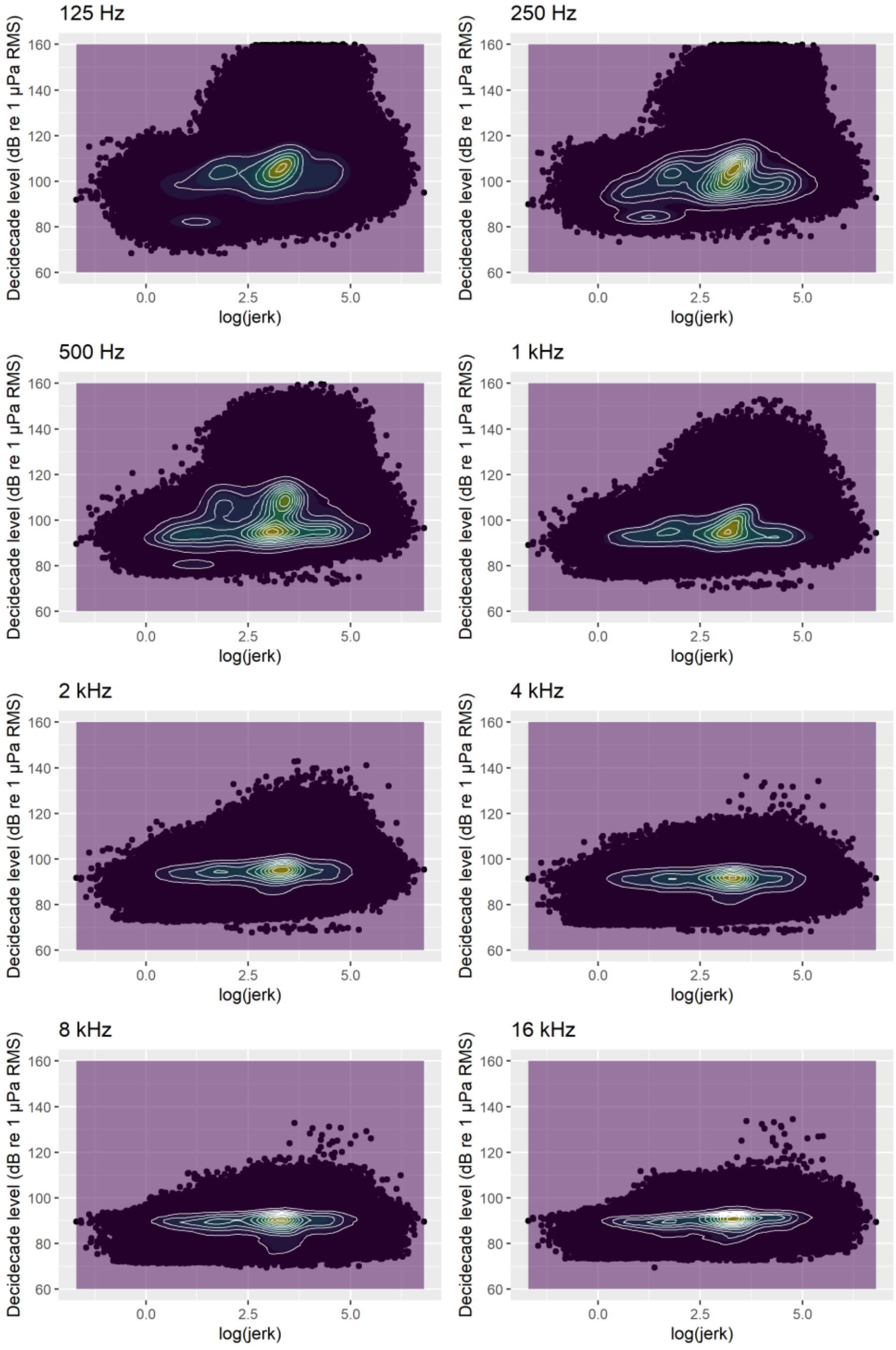

Supplementary Figure S2 (continued)

hs17_109b

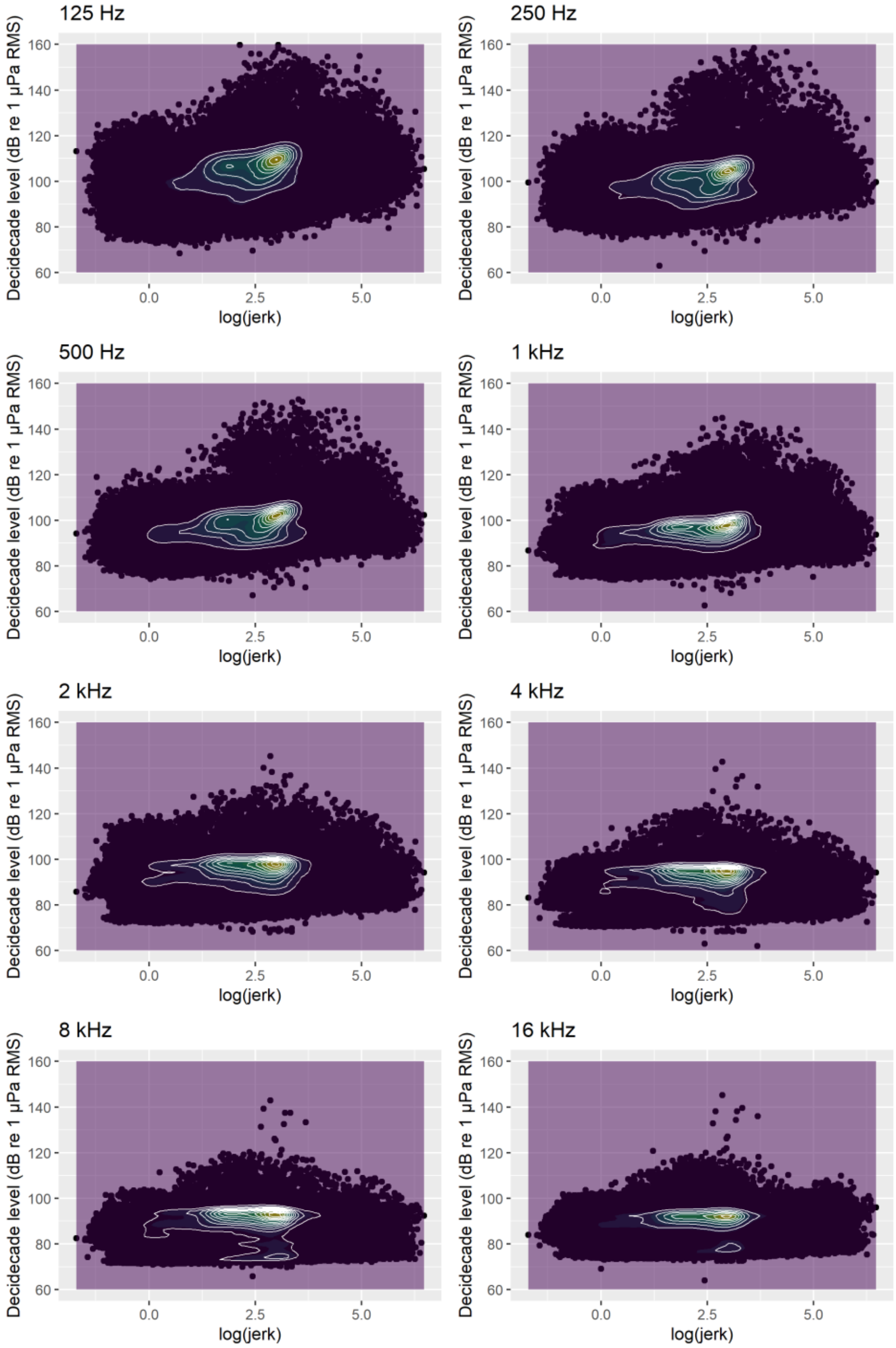

Supplementary Figure S2 (continued)

hs17_109c

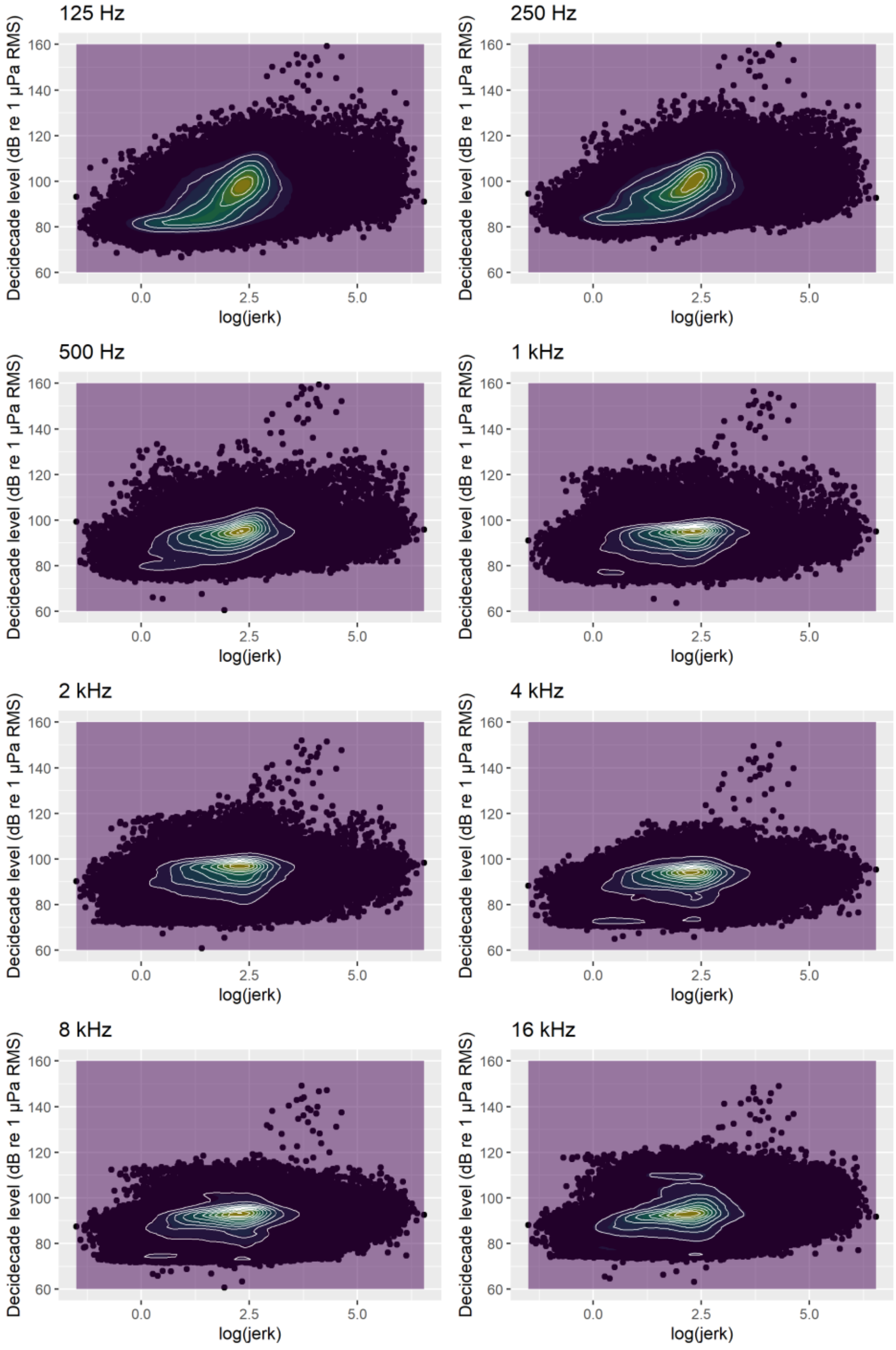

Supplementary Figure S2 (continued)

hs17_109d

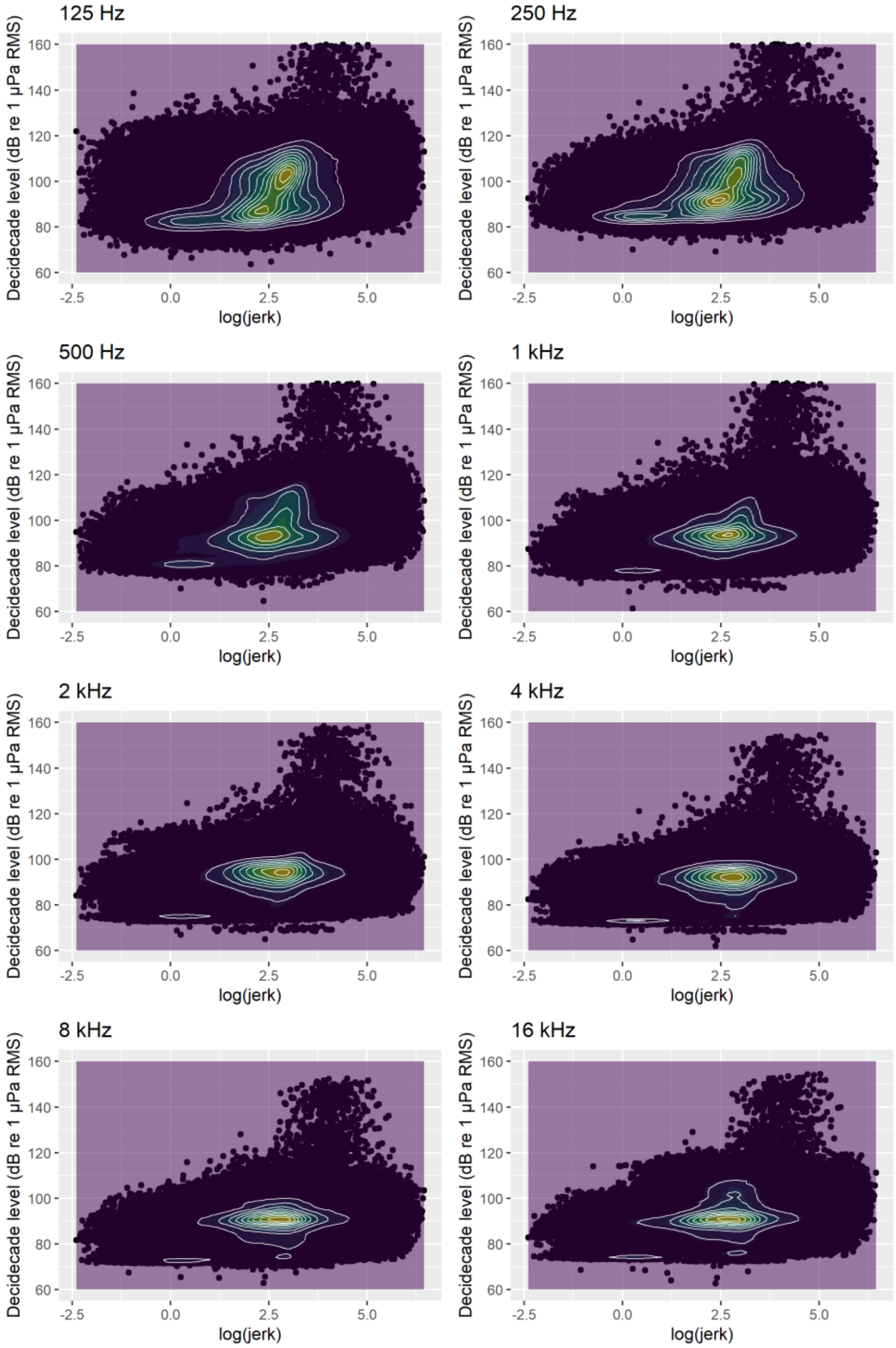

Supplementary Figure S2 (continued)

hs17_109e

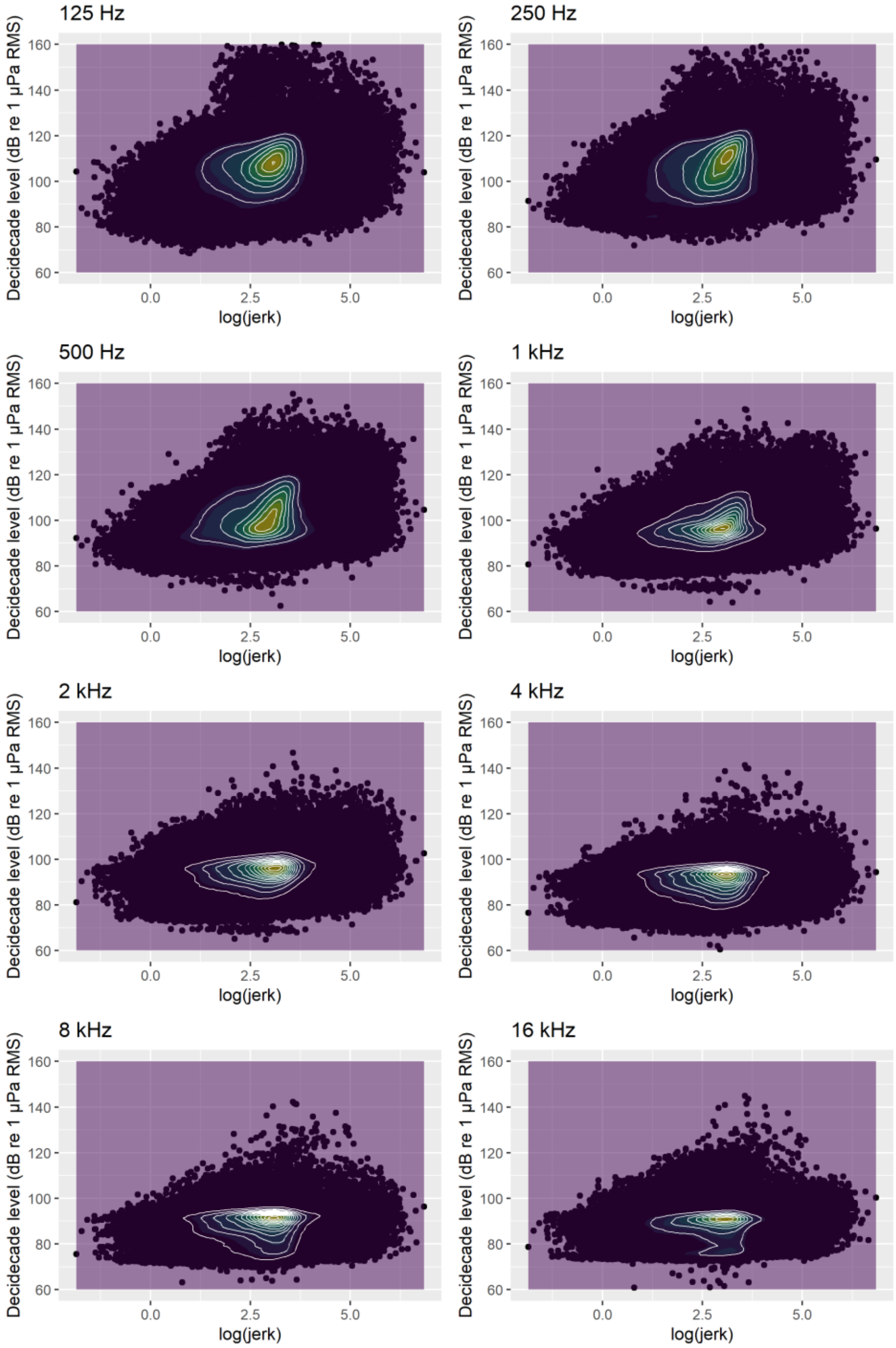

Supplementary Figure S2 (continued)

hs17_283a

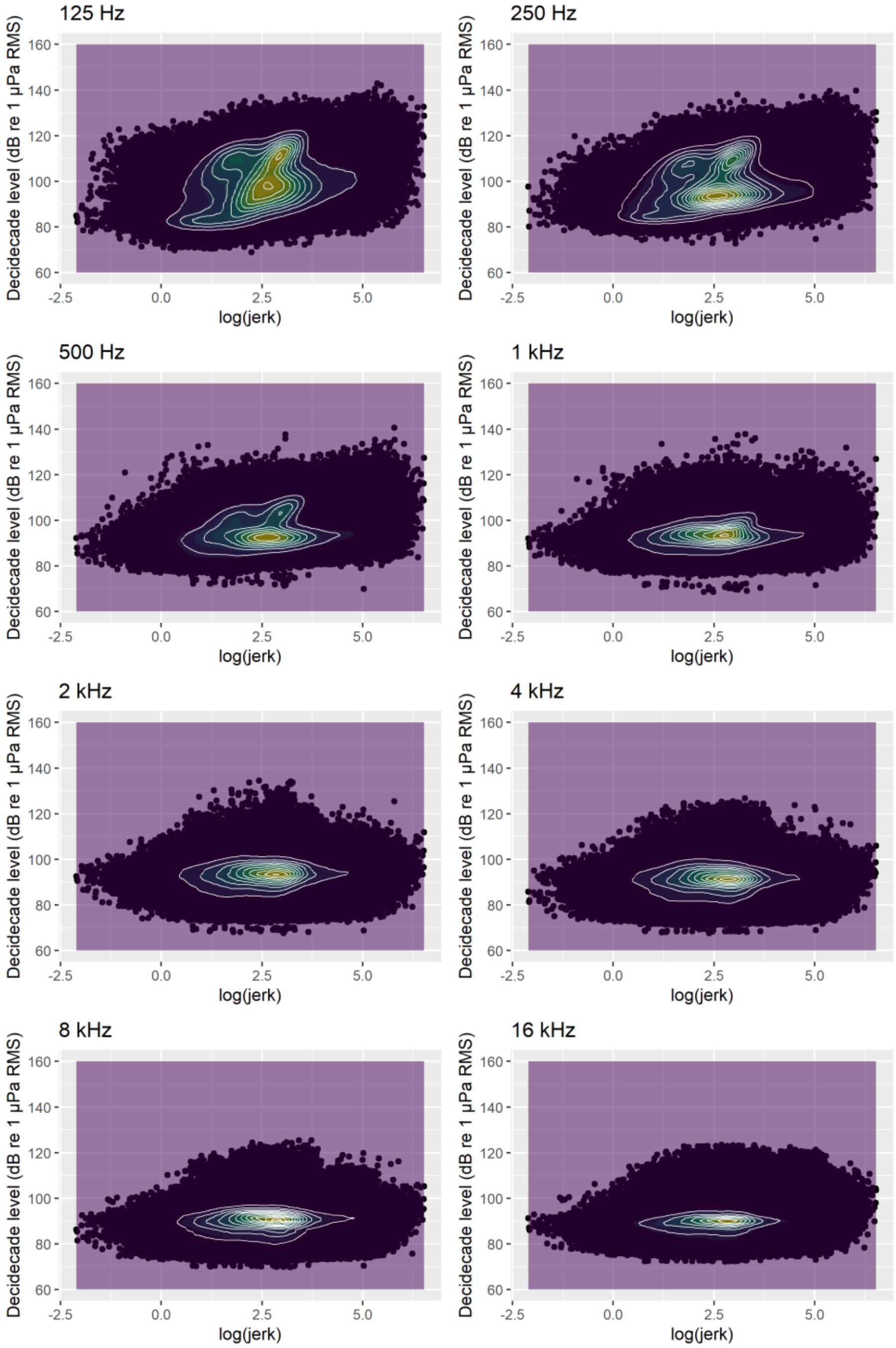

Supplementary Figure S2 (continued)

hs17_283b

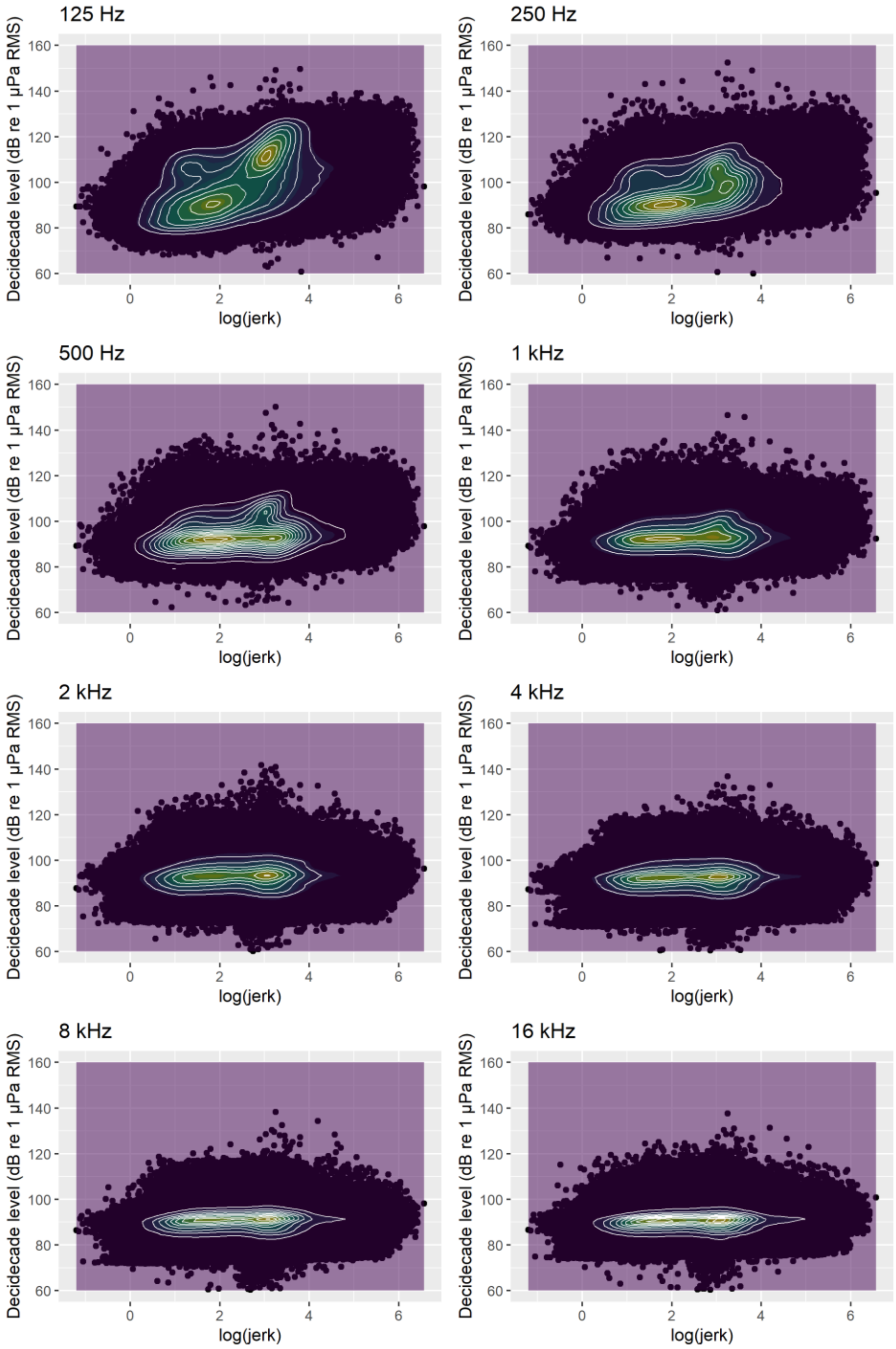

Supplementary Figure S2 (continued)

Supplementary Figure S3

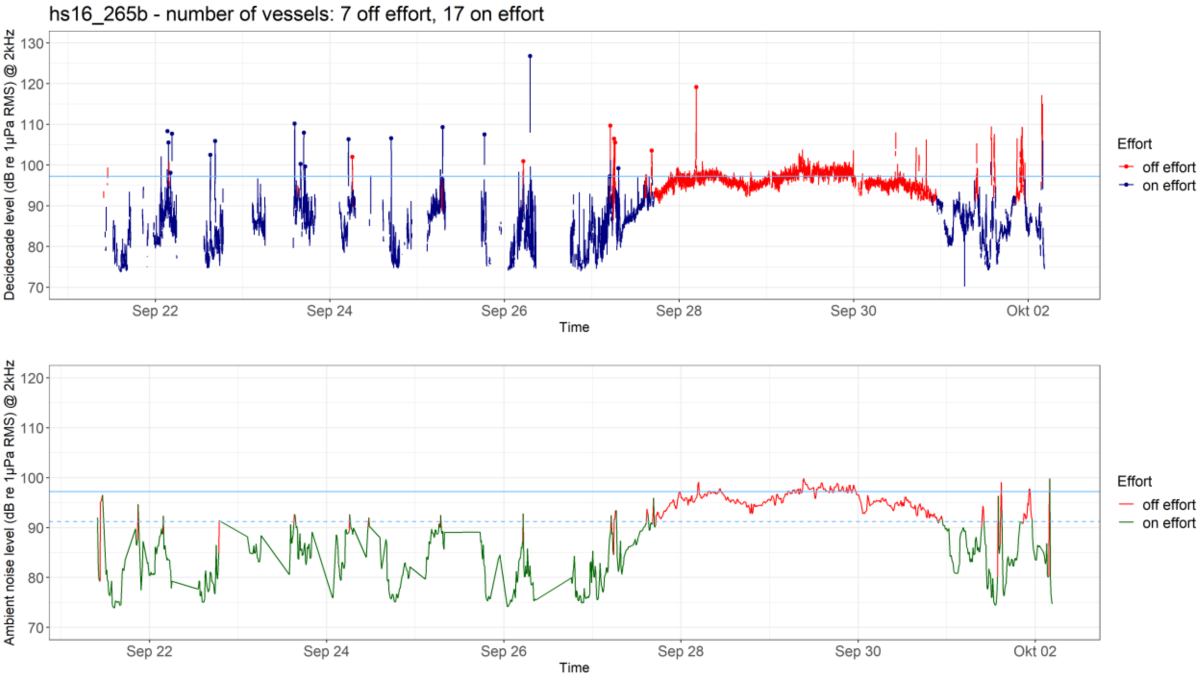

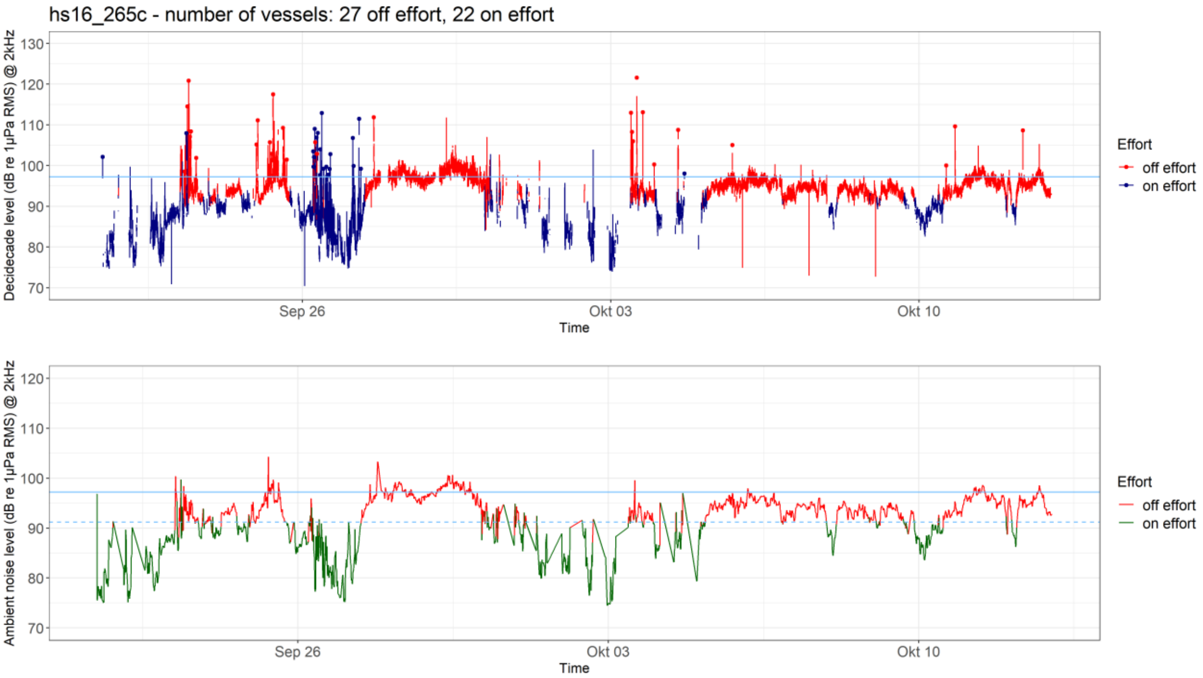

hs17_109a - number of vessels: 18 off effort, 6 on effort

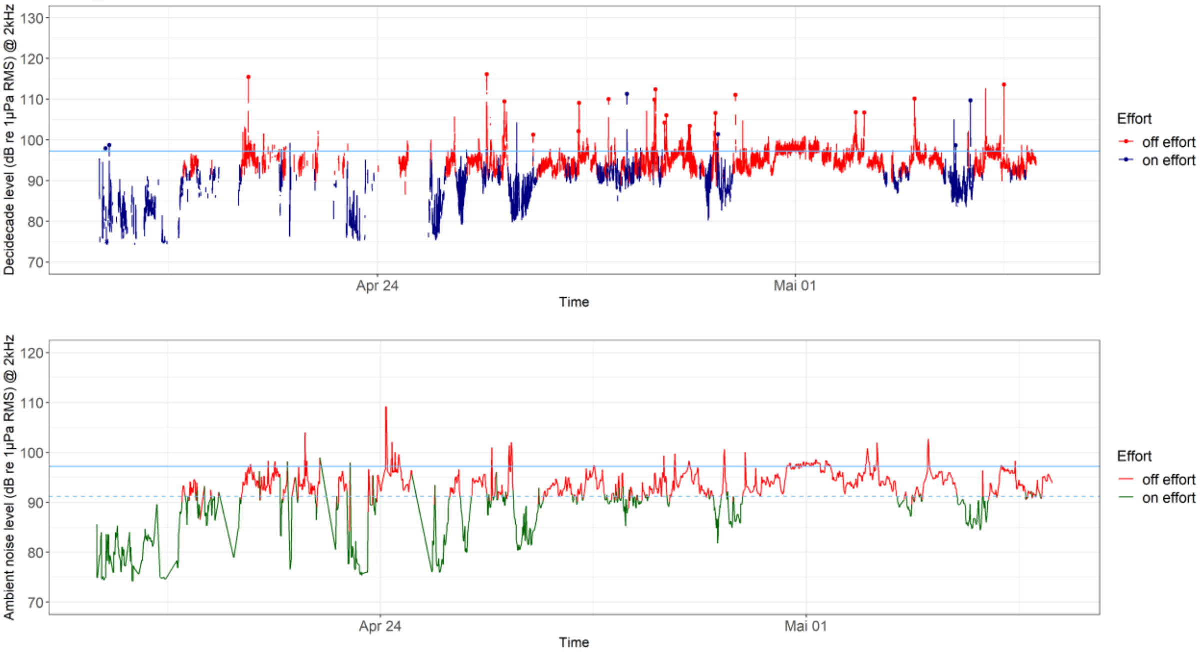

hs17_109b - number of vessels: 8 off effort, 4 on effort

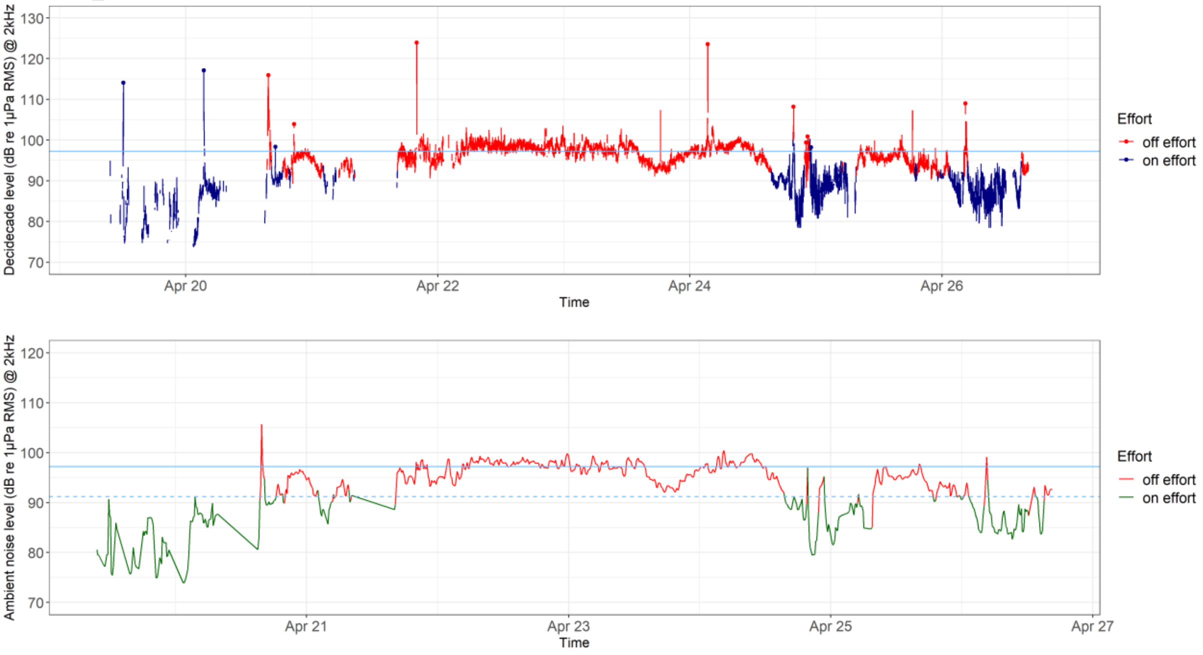

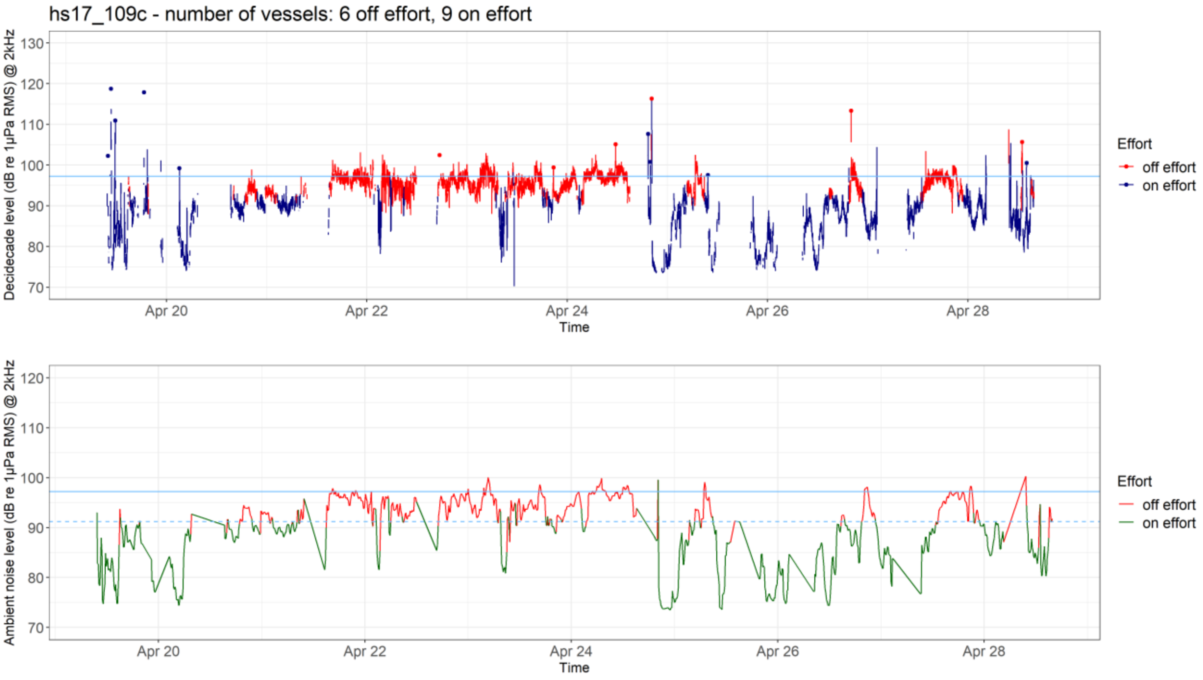
hs17_109c - number of vessels: 6 off effort, 9 on effort
Decidecade level (dB re 1µPa RMS) @ 2kHz
130
120
110
100
90
80
70
Apr 20
Apr 22
Apr 24
Apr 26
Apr 28
Time
Effort
off effort
on effort
Ambient noise level (dB re 1µPa RMS) @ 2kHz
120
110
100
90
80
70
Apr 20
Apr 22
Apr 24
Apr 26
Apr 28
Time
Effort
off effort
on effort

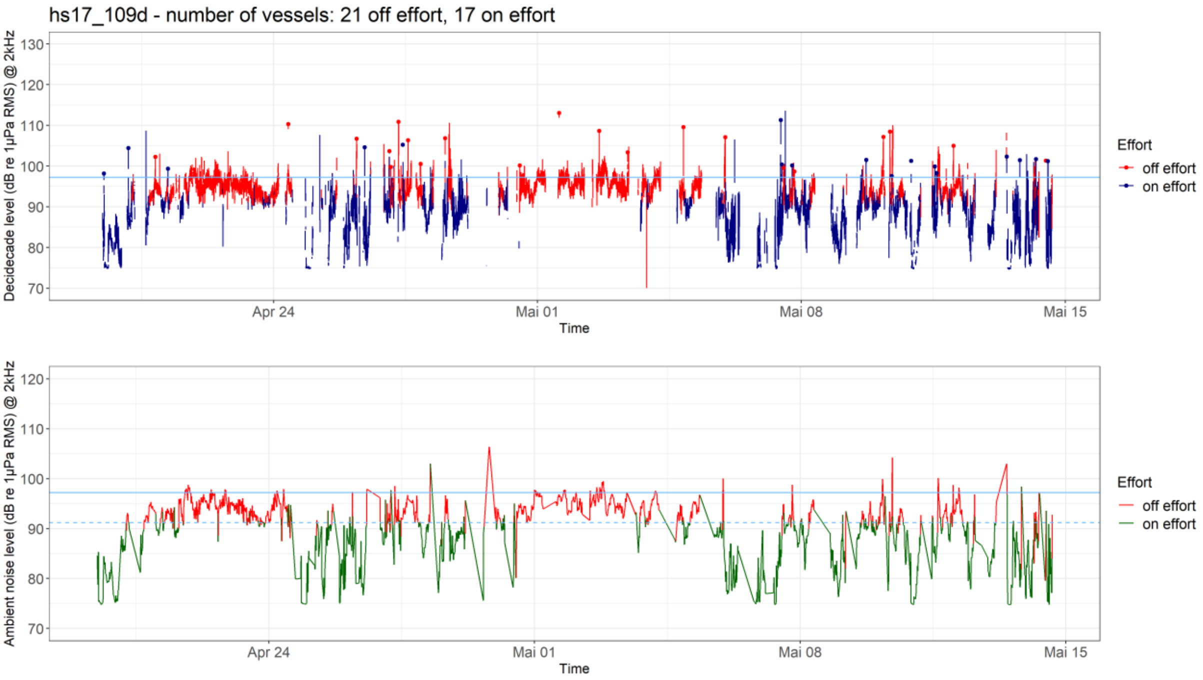
hs17_109d - number of vessels: 21 off effort, 17 on effort
Decidecade level (dB re 1µPa RMS) @ 2kHz
130
120
110
100
90
80
70
Apr 24
Mai 01
Mai 08
Mai 15
Time
Effort
off effort
on effort
Ambient noise level (dB re 1µPa RMS) @ 2kHz
120
110
100
90
80
70
Apr 24
Mai 01
Mai 08
Mai 15
Time
Effort
off effort
on effort

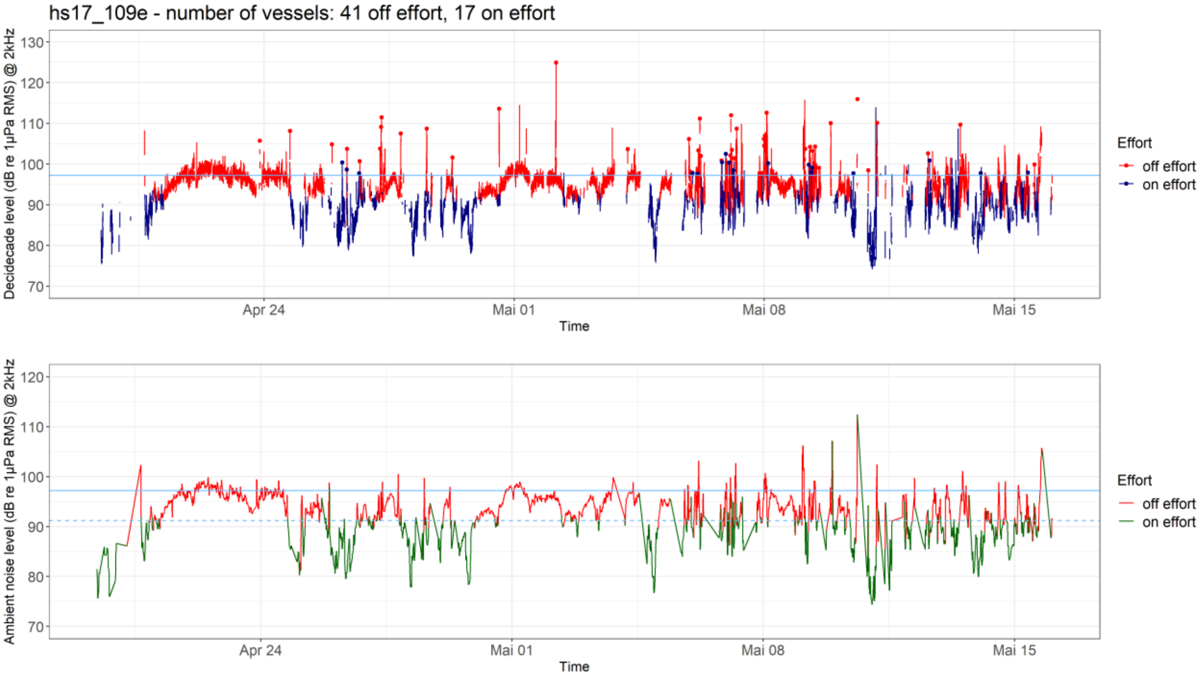
hs17_109e - number of vessels: 41 off effort, 17 on effort
Decidecade level (dB re 1µPa RMS) @ 2kHz
130
120
110
100
90
80
70
Apr 24
Mai 01
Mai 08
Mai 15
Time
Effort
off effort
on effort
Ambient noise level (dB re 1µPa RMS) @ 2kHz
120
110
100
90
80
70
Apr 24
Mai 01
Mai 08
Mai 15
Time
Effort
off effort
on effort

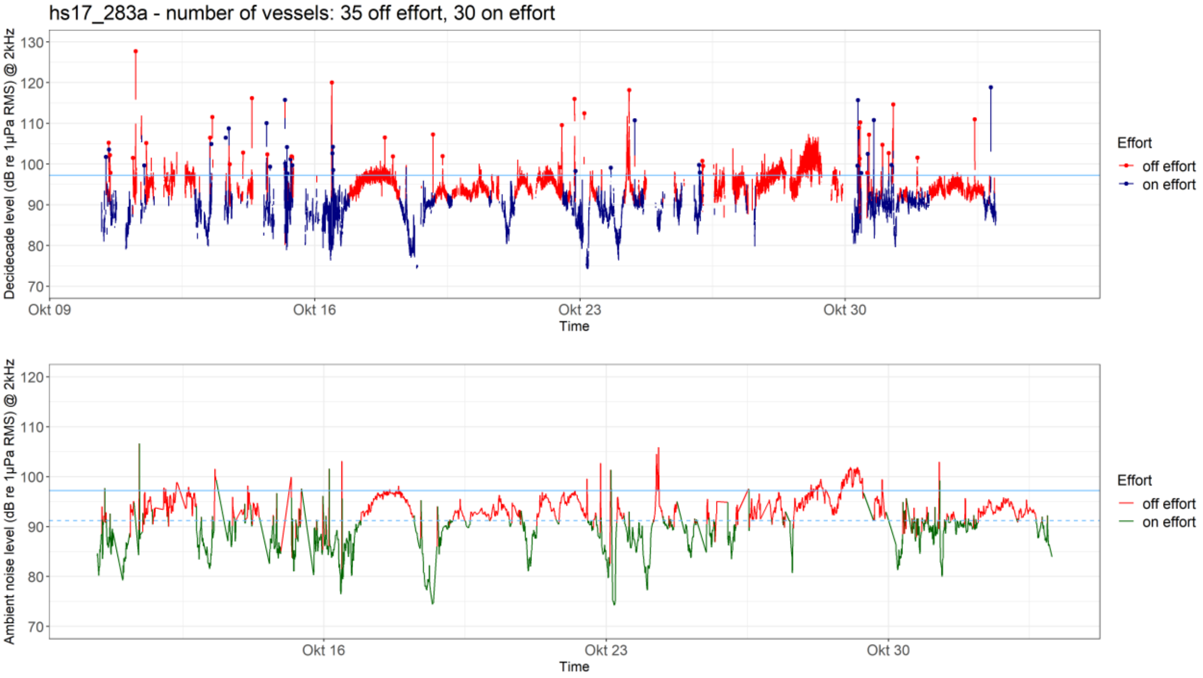
hs17_283a - number of vessels: 35 off effort, 30 on effort
Decidecade level (dB re 1µPa RMS) @ 2kHz
130
120
110
100
90
80
70
Okt 09
Okt 16
Okt 23
Okt 30
Time
Effort
off effort
on effort
Ambient noise level (dB re 1µPa RMS) @ 2kHz
120
110
100
90
80
70
Okt 16
Okt 23
Okt 30
Time
Effort
off effort
on effort

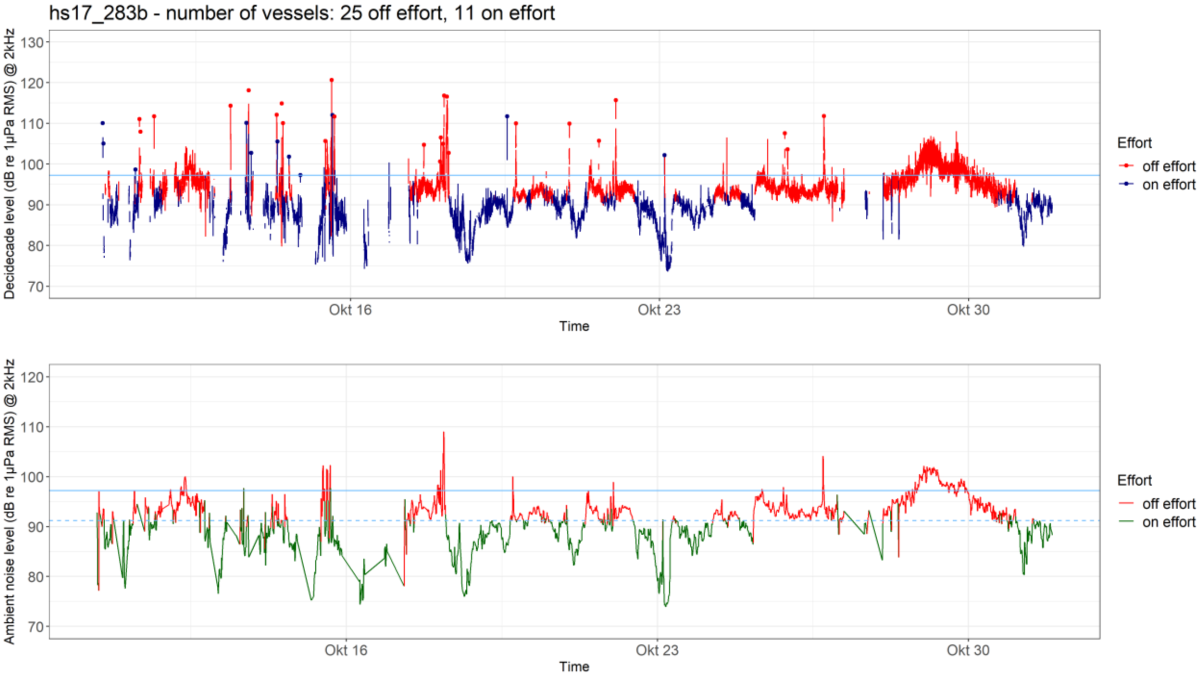

Supplementary Figure S3: Time series of 2 kHz decidecade band levels (<u>top image</u>) and inferred ambient noise levels (<u>bottom image</u>) for each seal. The 2 kHz decidecade band levels (dB re 1 µPa RMS) are computed as 30 s averages as described in the main text. The ambient noise levels were calculated from the 2 kHz decidecade band levels (dB re 1 µPa RMS) by taking the 25th percentile of the 30 s measurements over a 20 min interval with 10 min overlap. The solid light blue line illustrates the detection threshold of 97 dB re 1 µPa used to detect vessel passes. On-effort periods (dark blue and green) are defined as periods when the ambient noise levels were ≥6 dB below the detection threshold (dashed light blue line). Off-effort periods are shown in red and indicate periods with higher ambient noise levels during which some vessel passes might be missed. The off-effort periods were excluded for estimation of vessel encounter rates.

Supplementary Table S1

Supplementary Table S1: Translation of numeric AIS vessel codes into AIS ship types, taken from https://api.vtexplorer.com/docs/ref-aistypes.html (URL accessed on 27.02.2022). The third column shows the ship types as used in the present study with minor adjustments to the original classification. The fourth column shows the ship types used for the source level model following MacGillivray & de Jong (2021).

AIS vessel code	AIS ship type	Adjusted AIS ship type for this study	AIS ship type used for source level model
0	Not available or no ship	Not available or no ship	Not available or no ship
1 - 19	reserved for future use	Other Type	Other Type
20 - 29	Wing in ground (WIG)	Wing in ground (WIG)	Other Type
30	Fishing	Fishing	Fishing
31	Towing	Towing	Tug
32	Towing: length exceeds 200m or breadth exceeds 25m	Towing: length exceeds 200m or breadth exceeds 25m	Tug
33	Dredging or underwater ops	Dredging or underwater ops	Dredger
34	Diving ops	Diving ops	Other Type
35	Military ops	Military ops	Naval
36	Sailing	Sailing	Recreational
37	Pleasure craft	Pleasure craft	Recreational
38	reserved	Other Type	Other Type
39	reserved	Other Type	Other Type
40 - 49	High Speed Craft (HSC)	High Speed Craft (HSC)	Other Type
50	Pilot vessel	Pilot vessel	Other Type
51	Search and Rescue vessel	Search and Rescue vessel	Government / Research
52	Tug	Tug	Tug
53	Port Tender	Port Tender	Government / Research
54	Anti-pollution equipment	Anti-pollution equipment	Other Type
55	Law enforcement	Law enforcement	Government / Research
56	Spare - Local vessel	Spare - Local vessel	Other Type
57	Spare - Local vessel	Spare - Local vessel	Other Type

58	Medical Transport	Medical Transport	Other Type
59	Ship according to RR Resolution No.18	Ship according to RR Resolution No.18	Other Type
60 - 69	Passenger	Passenger	Passenger (length ≤100 m)<br>Cruise (length >100 m)
70 - 79	Cargo	Cargo	Bulker (70,75-79 speed ≤16 kn)<br>Containership (70,75-79 speed >16 kn, 71-74 all speeds)
80 - 89	Tanker	Tanker	Tanker
90 - 99	Other Type	Other Type	Other Type

Supplementary Methods: ‘Derivation of fixed detection threshold’

Prior to applying the methods described in the main text, vessel passes in the DTAG sound recordings were annotated using a supervised detector with an adaptive threshold. The aim of this initial assessment was to collect a wide range of vessel noise measurements from which to derive a fixed detection threshold that could be employed over the recordings of all seals. As with the fixed-threshold method described in the main text, the 2 kHz decidecade and an averaging time of 30 s were used for this analysis. The detection threshold was chosen as the 75th percentile of noise levels in each WAV file in the deployment (mean ± SD duration of WAV files: 5.0 ± 2.0 h). The use of a varying threshold does not allow the calculation of vessel noise exposure rates as the detection sensitivity varies with time. However, this method yields detections over a large range of received levels, from faint vessel passes (at low ambient noise conditions) to very loud vessel passes.

Each detection was subsequently validated as a vessel pass by examining the spectrogram and headphone listening to 1 min of the audio recording around the peak. This resulted in a collection of 491 vessel passes for ten harbour seals (nine seals included in the study plus one additional deployment, which was later excluded due to short deployment duration).

To derive a fixed detection threshold from these annotations, we compared the distributions of the 2 kHz decidecade levels (30 s averages) from all individuals as function of being during a vessel pass or not. We applied a cutpoint analysis (function *cutpointr* in the R package *cutpointr* with default settings (Thiele, 2021)) to determine the optimal cutpoint between both distributions (Supplementary Figure S4). The optimal cutpoint was determined as 97 dB re 1 µPa RMS @ 2 kHz corresponding to an Area Under the Curve (AUC) of 0.86, a sensitivity of 0.73 and a specificity of 0.87.

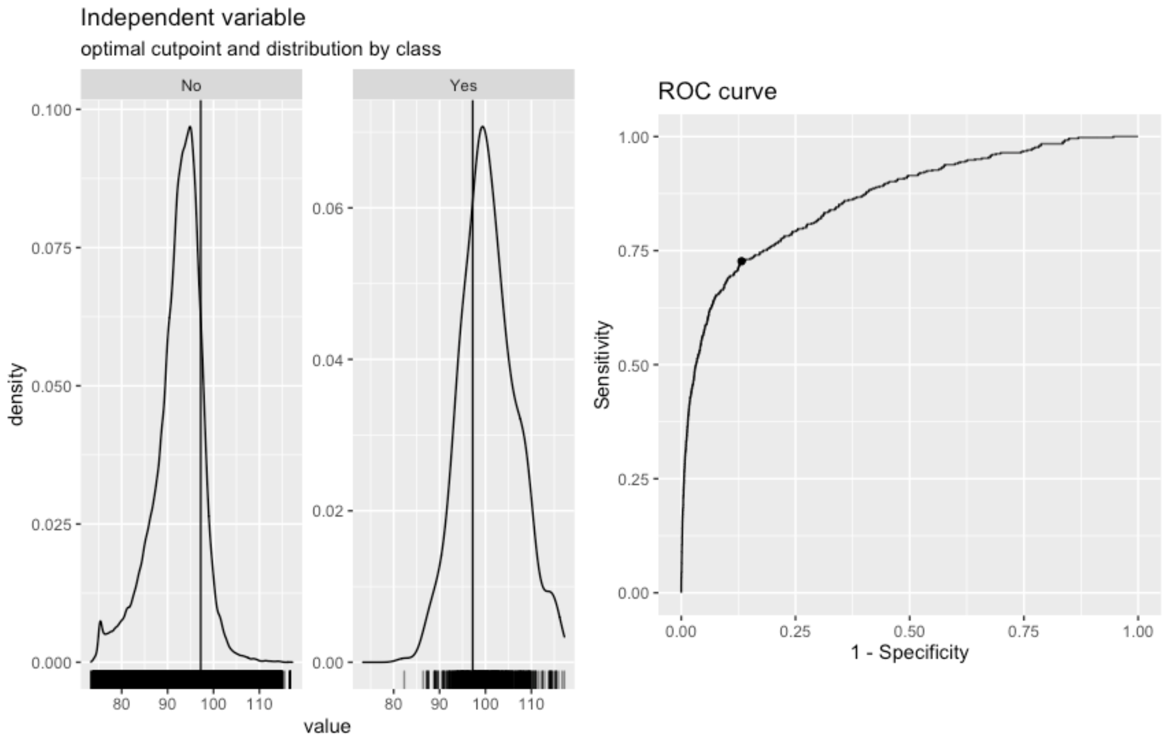

Supplementary Figure S4: Results of the cutpoint analysis. The two density plots on the left show the distribution of the 2 kHz decidecade levels at the peak of each vessel pass ('Yes') and the distribution at all remaining periods ('No'). The ROC curve shows the relationship between sensitivity and specificity and illustrates the optimal cutpoint.

References

Thiele, C. (2021). cutpointr: Determine and evaluate optimal cutpoints in binary classification tasks. R package 1.1.0. Retrieved from https://cran.r-project.org/package=cutpointr

Supplementary Methods: 'High noise event classification'

Classification of high noise events

Each high noise event, identified by the fixed-threshold detector described in the main text, was classified by three trained raters. Raters listened to, and inspected spectrograms of, the sound recording during each event. The raters could choose one of six categories as the source of each noise event (Supplementary Table S2). In case a rater classified the noise as 'vessel', they had to provide a reason for this decision (chosen from Supplementary Table S3). The raters examined the high noise events, pooled from the 9 seals, in a randomized order.

Supplementary Table S2: Categories for high noise events classification.

Category	Description
Vessel	Vessel noise, as defined by the cues in Table S3
Potential vessel	Potentially vessel noise, however, the noise characteristics do not match the cues in Table S3
Other anthropogenic	Other anthropogenic noise sources, e.g., acoustic deterrent devices or pile driving
Weather/rain	Noise due to rain or high wind speeds
Tag noise	Noise due to movement or sound production of the tagged animal, e.g., strong flow noise, hydrophone contact with seafloor, animal vocalisations
Unknown	Unknown noise source

Supplementary Table S3: Cues for the decision on vessel noise.

Cues	Description
Lloyd's Mirror	The Lloyd's Mirror Effect is caused by interference between sound waves travelling on the direct path to the receiver and sound waves reflected from the sea surface. This phenomenon produces distinctive U-shaped lines in the spectrogram for a moving source at a fixed depth.
Envelope	A visible and audible increase and decrease of noise levels as a result of a passing vessel.
Rotating machinery noise	The strongest noise source of a vessel is typically propeller cavitation. The resulting noise is often amplitude-modulated at the propeller blade passing rate, leading to a pulsing or rhythmic noise.
Whiney whoosh	In smaller vessels, the propeller blade rate can be very high, leading to a cavitation noise that is audible as a whoosh sound. The high propeller blade rate may also lead to a 'singing propeller', a narrow band noise caused by the vibrating propeller blades.
Echosounder	Most vessels are equipped with echosounders that emit repetitive pulses at a fixed frequency to measure the water depth below the vessel or, on fishing vessels, to locate aggregations of fish.

Multi-rater analysis

To quantify the agreement between raters when classifying high noise events, we used Cohen's kappa coefficient (*k*).

For agreement analysis, the six categories were unified into: 'vessel', 'potential vessel' and 'other', as the aim was to quantify the ability of raters to identify vessels compared to other noise sources. To estimate *k*, we used a weighted estimate, meaning that we weighted the

agreement/disagreement differently between categories. For the weighted kappa, we used a symmetric matrix with zeros in the diagonal, 0.5 for vessel vs. potential vessel, and 1 for vessel vs. other, i.e., a disagreement between raters in which one rater selects 'vessel' while another selects 'potential vessel' is weighted half as much as a complete disagreement in which the second rater selects a non-vessel sound category.

Results

A total of 560 high noise events were identified and classified from tag recordings on nine harbour seals. On average, the agreement between raters (Cohen's kappa) was 0.88, ranging from 0.74 to 1 (Supplementary Table S4).

Supplementary Table S4: Results of the Cohen's kappa analysis for each seal.

**Animal ID**	**Number of high noise events**	**Cohen's kappa *k***
hs16_265b	48	0.88
hs16_265c	66	0.91
hs17_109a	60	0.89
hs17_109b	14	1.00
hs17_109c	38	0.87
hs17_109d	101	0.84
hs17_109e	96	0.74
hs17_283a	76	0.88
hs17_283b	61	1.00

Of the 560 events, 321 (57.3%) were classified as vessels, 22 (3.9%) as potential vessels, and 210 (37.5%) as other noise events (Supplementary Figure S5). Events were classified as true vessels, if either all raters classified it as a vessel, or two raters classified it as a vessel and one

as a potential vessel. The raters completely disagreed on 7 events (1.2%) (Supplementary Figure S5).

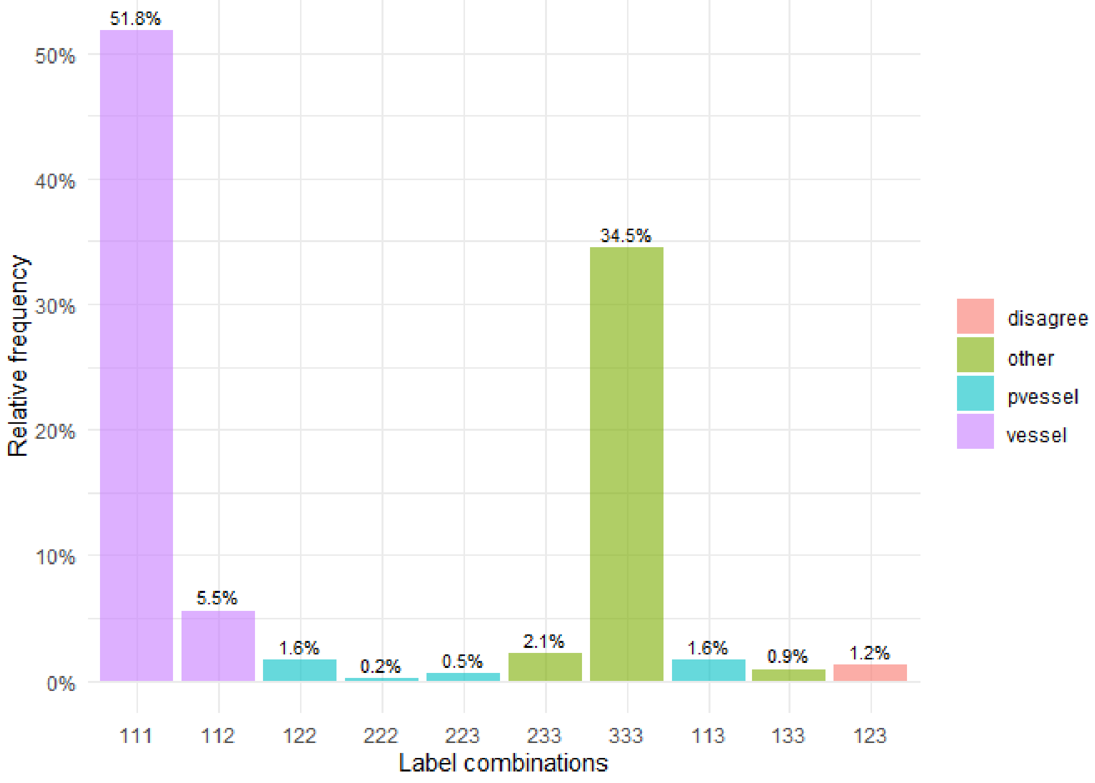

Supplementary Figure S5: Barplot of the relative frequency of classification decisions made by three raters. Each bar represents a classification combination of the three raters. 1 = 'vessel', 2 = 'potential vessel' and 3 = 'other'. For instance, combination 112 means that two raters classified the event as vessel, while one rater classified it as potential vessel. In the majority of cases (86.5%), the classification decision was unanimous (111, 222 or 333). The colours of the bars indicate the final classification decision for each event. For instance, events were only regarded true vessels, if either all raters classified it as a vessel or two raters classified it as a vessel and one as a potential vessel.

Supplementary Methods: 'Association between vessel noise exposures and AIS data'

The goal of the association analysis is to assess if any AIS-registered vessel present within 20 km could plausibly be the source of the vessel noise exposure. To accomplish this, we considered two goodness-of-fit indicators for each vessel pass and potential AIS vessel: (i) the time difference between the time of peak exposure on the tag and the estimated closest approach time of the vessel to the seal, and (ii) the shape of the noise exposure (i.e., the rise and fall rates of the sound level) compared to the predicted shape given the speed and distance of the vessel.

The first of these indicators requires an estimate of the closest approach time of the vessel to the seal, and this was inferred from the interpolated position of the seal at peak exposure (see below Supplementary Methods: 'Calculation of seal locations during the peak time of each vessel pass') and the GPS positions of the vessel reported via AIS. The second indicator requires a parameterization of the shape of the noise exposure. To derive a suitable parameter, we first express the distance between the seal and the vessel as a function of time relative to the time of closest approach, $\tilde{t}$. Assuming that the seal is effectively stationary compared to the vessel and that the vessel travels on a straight course, the separation distance in metres is:

$$d = \sqrt{c^2 + \tilde{t}^2 v^2} \quad \text{(S1)}$$

where c is the closest approach distance in metres and v is the speed of the vessel in m/s. Assuming spherical spreading, the received level of the vessel noise, RL (dB re µPa RMS) is:

$$RL = SL - 20\log_{10}(d) \quad \text{(S2)}$$

where SL is the equivalent source level of the vessel (dB re µPa RMS at 1m). Substituting (S1) into (S2) and re-arranging gives:

$$RL = SL - 10\log_{10}(c^2 + \tilde{t}^2 v^2)$$

$$= SL - 10\log_{10}(c^2\left(1 + \frac{\tilde{t}^2 v^2}{c^2}\right))$$

$$= SL - 20\log_{10}(c) - \ 10\log_{10}\left(1 + \frac{\tilde{t}^2 v^2}{c^2}\right) \quad \text{(S3)}$$

Further rearranging gives:

$$10\log_{10}\left(1 + \frac{\tilde{t}^2 v^2}{c^2}\right) = RL - SL + 20\log_{10}(c) \quad \text{(S4)}$$

SL - $20log_{10}(c)$ is the estimated received level at closest approach, i.e., the peak RL of the exposure, RL_{pk}. Substituting this and taking the exponent of both sides to remove the logarithm gives:

$$1 + \frac{\tilde{t}^2 v^2}{c^2} = 10^{\frac{RL - RL_{pk}}{10}} \quad \text{(S5)}$$

This can be further simplified by combining the constants c and v into a single parameter, γ = c/v, i.e.:

$$1 + \frac{\tilde{t}^2}{\gamma^2} = 10^{\frac{RL - RL_{pk}}{10}} \quad \text{(S6)}$$

This formula indicates that the received level during a vessel pass, relative to the peak received level, and expressed as a power, should be a quadratic function of time, $\tilde{t}$. The parameter γ controls the shape of this quadratic: a small value of γ gives an exposure with a fast rise and fall time while a large γ leads to a received level that increases and decreases slowly. This shape parameter, γ, can be estimated in two ways: (i) by fitting a quadratic function of time (relative to peak exposure) to the observed received levels using (S6), γ_n; and (ii) by estimating c and v from the AIS reports of the target vessel, γ_v. If this vessel is the one causing the exposure, then the two methods for estimating γ should give similar values. Conversely, a mismatch between the two estimates of γ suggests that the vessel is not the cause of the exposure. This approach can be used to test each plausible vessel associated with a given exposure and eliminate those vessels that would produce an exposure with a very different shape than the one observed.

As γ is the ratio of c (in metres) and v (in metres/second), it has units of seconds and so relates to the rise/fall time of the received level as a vessel passes. Specifically, γ is the time needed for the RL to increase 3dB just before peak exposure or to fall 3 dB after peak exposure (this interpretation is evident in equation S6: when $\tilde{t}$ = γ, the predicted received power is one half

of the peak power, i.e., -3 dB in decibels). Critically, this parameter can be estimated independently using the sound recordings and using the AIS data making it possible to test the goodness-of-fit of each AIS-reported vessel to the exposure experienced by the seal. The derivation of γ involves several assumptions that may not be strictly met (e.g., spherical spreading) and we do not therefore expect a precise match between the values of γ computed from the received levels and from AIS. However, widely differing values shed doubt on a vessel being the source of the exposure.

To implement the vessel association method outlined above, γ_n was estimated for each detected vessel pass from the DTAG sound data with the following steps. First, the power in the three decidecade bands around 2 kHz was summed to get the 2 kHz octave level. This broader-band received level measure is more robust to interference and Lloyd mirror effects. The time of the maximum received level was then identified and all 2 kHz octave RL estimates within 5 minutes on either side of the peak were extracted, analogous to the time window in the AIS data analysis. These were processed to compute the attenuation factor relative to peak exposure using the formula:

$$a = 10^{RL-RL_{pk}}$$

A quadratic function was fit between $\tilde{t}$ and a, and γ_n was estimated as the inverse square-root of the first polynomial coefficient (i.e., the coefficient of $\widetilde{t^2}$) (Supplementary Figure S6).

The goodness-of-fit of the quadratic function was assessed by (i) evaluating the shape of the curve and (ii) calculating R^2, the coefficient of determination. In a few cases, the quadratic function predicted an inverse shape of the curve, leading to imaginary numbers for γ_n. These vessel passes were excluded for the association with AIS vessels. Furthermore, the distribution of R^2 was assessed and all vessel passes with an $R^2 \geq 0.7$ of the quadratic fit were deemed acceptable (Supplementary Figure S7).

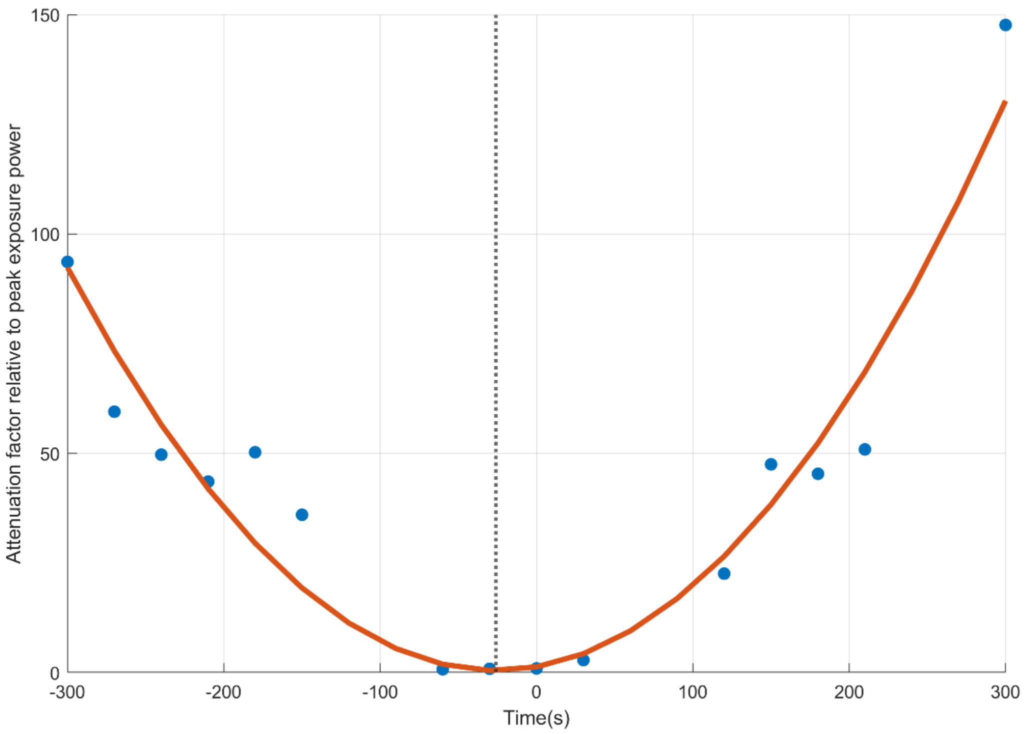

Supplementary Figure S6: Fit of a quadratic function (orange curve) to 2 kHz octave power measurements (blue points), normalised relative to the peak exposure power at time 0 using equation S6 (R^2 = 0.92). The time window covers 5 min before and after the peak exposure time, i.e., the time of maximum received levels.

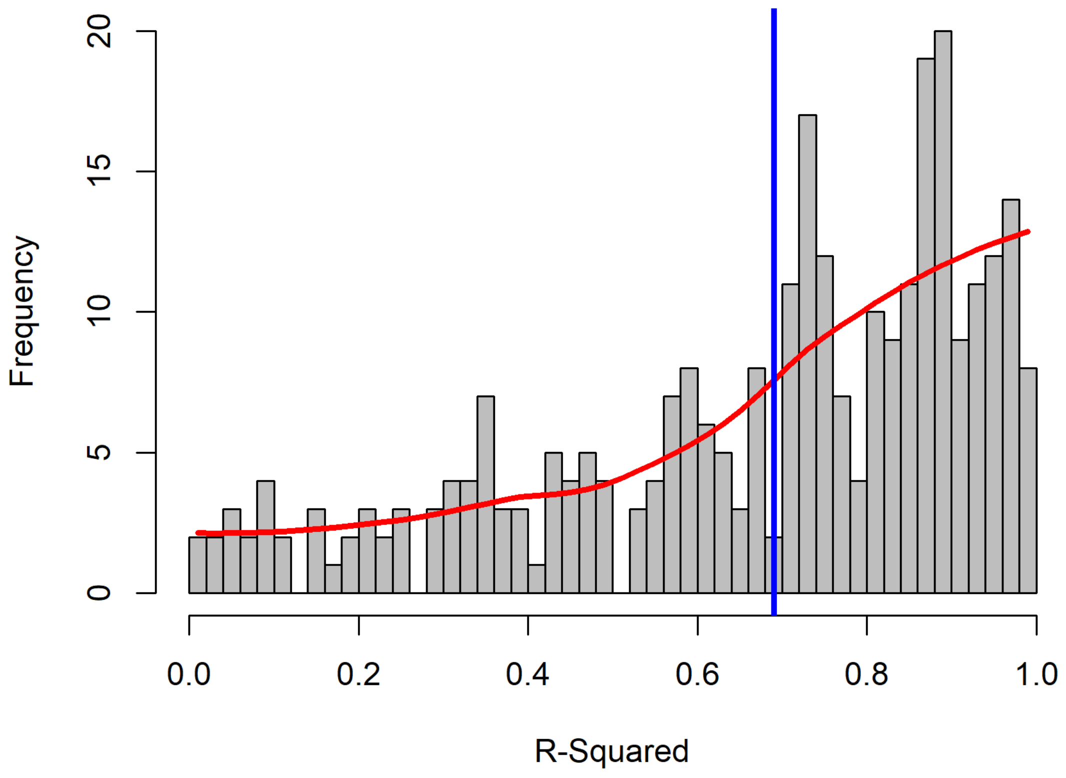

Supplementary Figure S7: Distribution of R^2 as a measure of the goodness-of-fit of the quadratic fit to estimate γ_n. The red curve is a loess smoother (span = 0.75) over the histogram bins. The blue vertical line illustrates the inflection point of the loess curve ($R^2 = 0.7$) and was chosen as the threshold to define an adequate fit of the quadratic function.

The γ parameter was also estimated from the AIS data by first computing the distance between the seal and each AIS vessel in a 20 km radius. Then, the speed of each vessel was estimated by the median reported speed over all AIS reports within the ± 5 min time period. γ_v was calculated by dividing the closest approach distance (in metres) by the speed of the ship (in metres per second).

As an initial assessment, we assumed that the nearest AIS vessel was the actual source vessel for each exposure and performed a linear regression between the γ values measured from the sound recording and from the AIS reports of the closest-approaching vessel. As the acoustically-derived γ_n values ranged from 0 to 400 seconds, we limited the regression analysis to γ_v values in the range 0-400 seconds. The resulting linear model ($n = 49$, $R^2 = 0.28$, $p < 0.001$, Supplementary Figure S8) was then used to test the γ_v value of all AIS vessels and thereby

identify which AIS vessels were most likely the source vessels (Supplementary Figure S9). Specifically, AIS vessels with a γ_v within ±2 standard deviations of the predicted value from the regression analysis were considered likely source vessels. If more than one such vessel was found for a given exposure, the best fitting vessel was taken as the most likely. Vessels with γ_v outside of this range were considered as unlikely source vessels as they would produce an exposure with very different rise and fall rates.

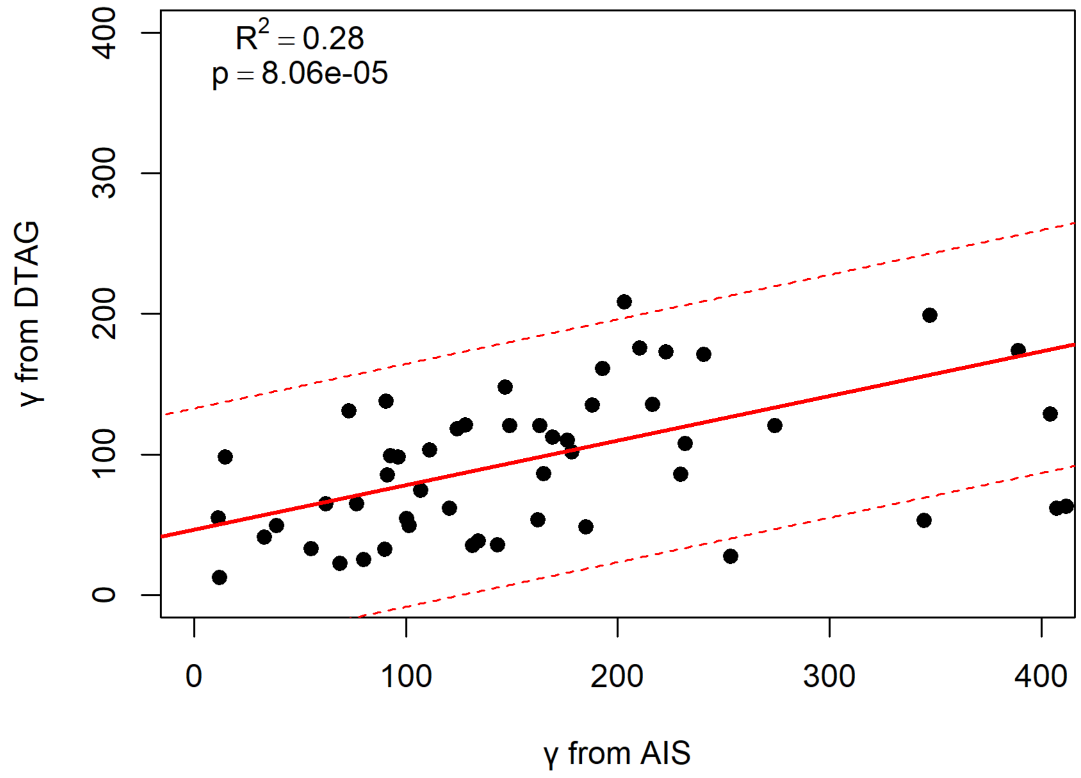

Supplementary Figure S8: Scatterplot (n = 49) of γ_v derived from the AIS data of the nearest vessel for each exposure vs. γ_n derived from the DTAG sound data. The γ_v values based on the AIS data (x-axis) were limited to values from 0-400. The red solid line shows the fit of the linear model, while the red dashed lines show two times the standard deviation of the model.

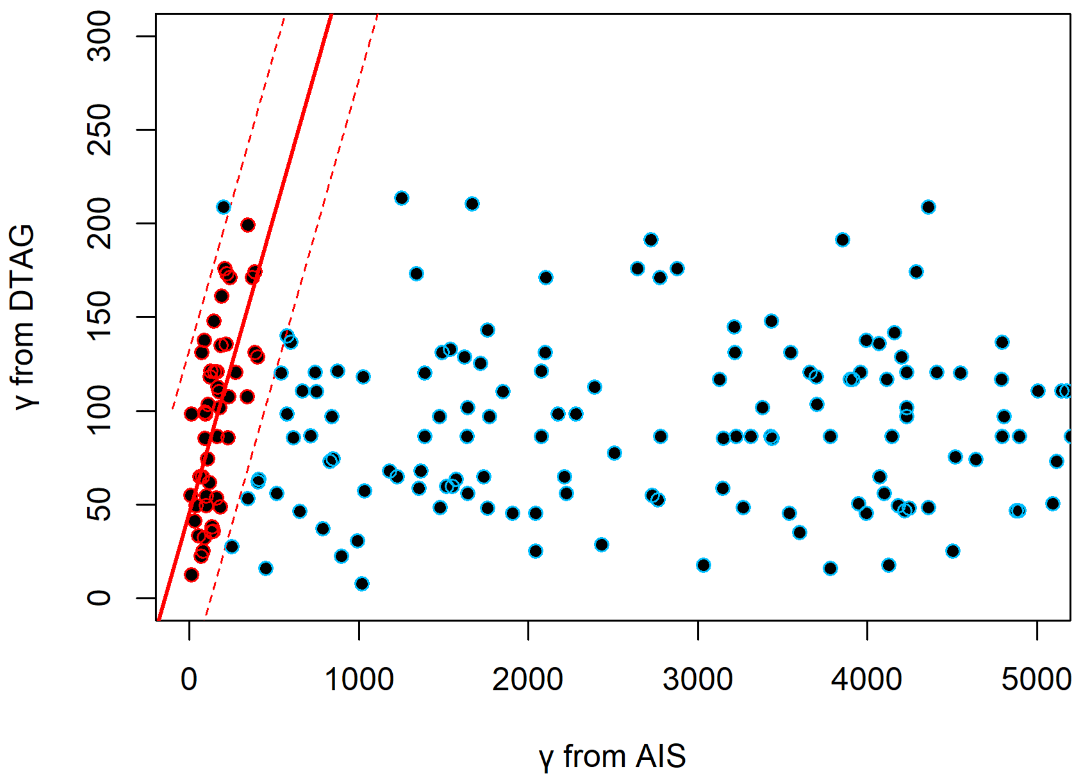

Supplementary Figure S9: Scatterplot of γ_v derived from the data of all AIS vessels vs. γ_n derived from the DTAG sound data. The data range on the x-axis was limited to values from 0-5000 (total range: 0 - 47,000). The red solid line shows the fit of the linear model, while the red dashed lines show 2 times the standard deviation of the model. Red points lie within the accepted range and hence represent a likely association between the recorded noise exposures and an AIS-reporting vessel. The blue points lie outside the accepted range and represent AIS vessels that are unlikely to be the source vessels. All but one of these unlikely vessels had an AIS-derived γ_v that was considerably larger than the γ_n estimated from the sound recording. This is consistent with the expectation that the closest passing vessel (which will typically have the lowest c/v ratio and therefore the lowest γ) is the most likely source vessel.

Supplementary Methods: 'Calculation of seal locations during the peak time of each vessel pass'

The GPS in the tags was programmed to sample position at 3 min intervals, but the actual sampling time depends on when the animal comes to the surface. Occasional long gaps in GPS acquisition occur when seals swim rapidly and make only brief surfacings, or when the sea-state is high. Linear interpolation of GPS positions was used to estimate the location of the seal during the peak time of each vessel pass. The time lag between the peak time and the nearest of the two bracketing GPS points was determined. The median time difference was 4 min; however, in a few cases the difference was higher, with a maximum of 617 minutes. To avoid large errors in the estimated position, we only performed linear interpolation if the nearest GPS point was less than 60 min away from the peak time. Assuming a typical horizontal speed of harbour seals of 1.3 m/s (4.7 km/h) as seen in the present study, this would result in a maximum deviance of 4.7 km from the closest known GPS position. However, an error this large would only occur if the seal swam in a completely different direction than that indicated by the bracketing GPS positions during much of the 60 min interval.

Of the 321 vessel passes (133 on-effort and 188 off-effort vessel passes), 61 were excluded from positioning analysis due to infrequent GPS positions. In the remaining interpolated locations, the median time difference to the nearest GPS point was 3 min. The median spatial distance between the interpolated seal locations and the closest GPS location was 101 m (min: 0, max: 3,289 m).

Supplementary Methods: ‘Ship sound propagation loss to determine maximum radius for AIS vessel association’

The goal of the vessel association analysis was to determine which, if any, of the AIS-reporting vessels in the vicinity of a seal was the most probable source of each above-threshold noise exposure in the tag recording. To simplify this analysis, we first estimated the maximum distance that a vessel could be from the seal and still produce an above-threshold exposure. This distance was estimated based on predicted source levels from different AIS ship types, followed by a simple sound propagation model. Source levels were computed for each ship type as spectrum levels following MacGillivray & de Jong (2021) and then converted into decidecade levels. Using the 2 kHz decidecade source level, a 15 $\log_{10}$ (Range) sound attenuation model was applied, reflecting a compromise between cylindrical and spherical spreading as seals encountered vessels over a range of water depths in the study area. The maximum distance, at which the modelled received level from vessels could be above the detection threshold of 97 dB, was found to be 20 km (Supplementary Figure S10).

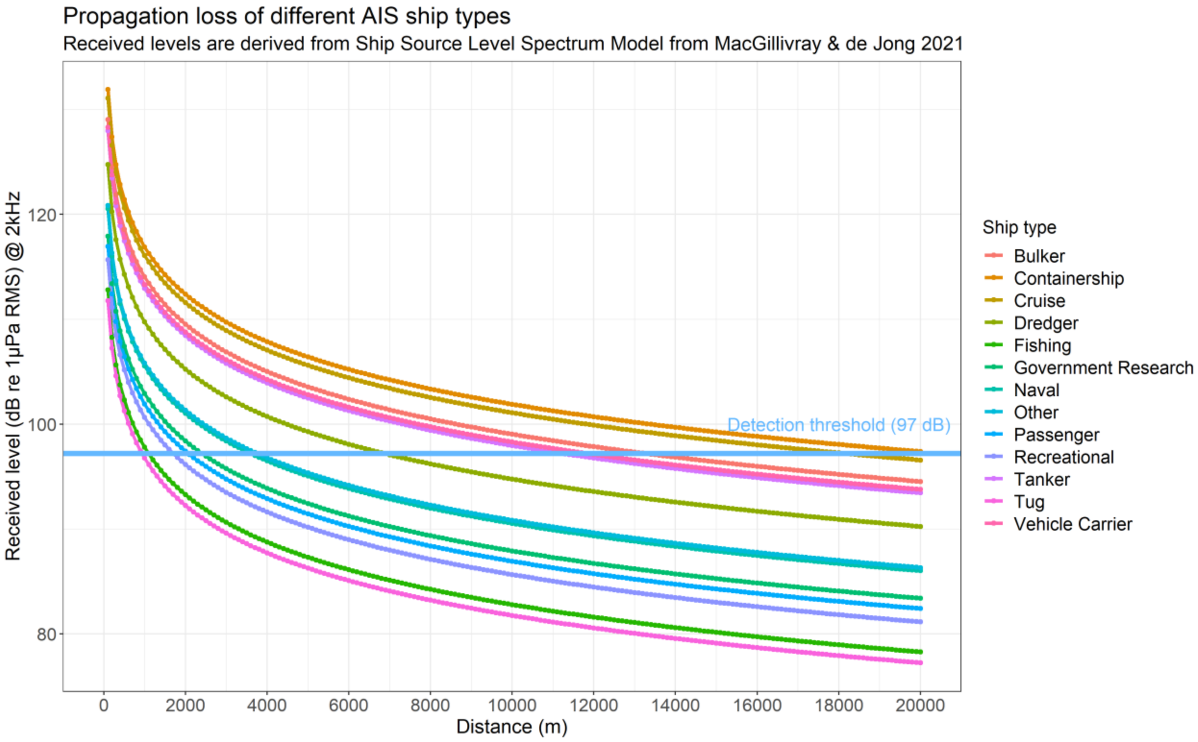

Supplementary Figure S10: Estimated received levels (RLs) of vessel noise in the 2 kHz decidecade band as a function of distance for different AIS-equipped ship types. Source levels

(SL) were determined (assuming average ship type lengths and speeds), using the Ship Source Level Spectrum Model described in MacGillivray & de Jong (2021) and then converted to decidecade levels. Corresponding RLs were calculated using a hypothetical propagation loss of 15 $\log_{10}$ (R), where R is the range from the sound source in metres. The maximum distance at which the received levels coincide with the detection threshold is 20 km (for containerships).
